# Analectic

**Published:** 1830

**Authors:** 


### Miscellaneous Intelligence.

### ANALECTIC, ANALYTICAL, AND ORIGINAL.

### I. Analectic.

1. *Incontinence of Urine cured by an Operation.*—Communicated to the London Medical and Physical Society, by Mr. Brodie.

Mary Conner, a respectable young woman, æt twenty, laboring under incontinence of urine, consulted me some time since, in the expectation of receiving some relief from symptoms which rendered her existence miserable. She gave me the following statement of her case:—That she had fallen down stairs about three years before, and had injured her back in the fall; from which period, up to the time of placing herself under my care, she gradually lost all command over the contents of the bladder; she had previously taken the advice of many professional men in Cork and elsewhere, without receiving any benefit, and, at the time above alluded to, suffered extremely from excoriation of the labia, and inside of the lower extremeties, from the constant flow of urine. The opinion entertained of her case was, that the symptoms were caused by paralysis of the neck of the bladder, produced by the fall, and that little or nothing could be done for her relief.

On examination, I found the urethra so much enlarged (so that, unless the power of retaining the urine depended entirely on the sphincter vesicæ, no check could be given whatsoever, to its constant flow) as to admit with facility a full sized rectum bougie. The sides of the canal lay quite loose and flabby, appearing to have entirely lost the power of contractility. * * * Every plan that could be devised, and every medicine that could be thought of, were employed, but without producing the slightest improvement. I then, a second time, proposed my former plan of treatment, which was agreed upon, and the operation was performed in the following manner, in the presence of my friends, Drs. Pitcairn and Bull.

Having placed her on her knees on a table, with her head downwards, and dilated the vagina with Weis' speculum, I introduced the index finger of my left hand into the urethra as far as the neck of the bladder, and having passed a sharp-pointed bistoury to the end of my finger, then raising its point, and cutting through the side of the urethra, so as to form one cavity of that canal and the vagina; I removed a portion of the urethra, the entire length of the canal, and of a triangular shape, with a knife-edged scissors. The lips of the wound were then brought together by means of pins, and secured by the interrupted suture.

The pins were all removed at the end of eight days, leaving the parts perfectly united, and the woman completely restored, having entirely recovered the perfect control over the discharge of urine.—The urethra at this time has to all appearances recovered its natural tone and size.—*Lond. Med. and Phys. Jour.*

2. *Recovery from Ruptured Uterus.*—Mrs. Cuthbert, aged 32, was taken in labor whilst on a journey through this town, on the evening of the 6th August last. The full period of gestation was completed, but notwithstanding her advanced state of pregnancy, she had travelled upwards of 200 miles on foot, within a very short period. On her arrival here she called in the aid of a female practitioner, who remained with her during the night of the 6th, and who described the presentation to have been natural, and the labor forward and rapid in progress. This person stated, that during a pain, from which she expected the immediate expulsion of the head of the child, the patient at the time on her feet by the side of the bed, a gush took place, the head of the child receded, and the labor pains ceased entirely. This took place about 5 o'clock in the morning of the 7th. About 7 o'clock, I was called, and found the poor woman in great distress. The floor of the small apartment had been flooded over. Drops of cold sweat stood on the patient's face, which was ghastly and anxious. She complained of constant "tearing pain." Her breathing was labored and oppressed. Her pulse 130, small, and feeble. She was vomiting dark-colored fluid. The short history which I obtained of the case, at once convinced me of its nature. On laying my hand on the abdomen, the form of the child could be distinctly felt through the parietes. On examination, I found the *os uteri* rigid, and so closely contracted, as hardly to admit the point of my finger. Passing my hand forward to the right side of the cervix, I readily ascertained the commencement of the rupture, which extended towards the fundus. The child was high up in the abdomen. Under these circumstances I thought it advisable to summon further aid, and Mr. Dalton, an able and experienced surgeon of this town, soon joined me. That gentleman at once agreed with me as to the nature of the mischief. On introducing his hand, he found the laceration to be as I have described it; and whilst tracing its extent, he fortunately came in contact with one of the child's feet. He soon got hold of the other, and brought both down. The extraction was proceeded with gradually and firmly; but the great difficulty was found to consist in bringing the head through the outlet of the pelvis. The pelvis was of contracted dimensions; and I learned that her former labors, two or three in number, had been attended with great difficulty. We found the obstruction to proceed from the superior maxillary bone of the left side, being fixed on the symphisis pubis. The head of the child thus lay obliquely in the pelvis; the right ear nearly opposite the sacrum, and the chin and mouth being the nearest presenting parts. We tried, by fixing the fingers on the lower jaw, to place the head in a better position, or to extract it, without success. Perforation through the basis of the cranium could, in this case, we considered, little facilitate its passage. We found the long forceps of little use, it being difficult to fix them properly. The vectis was applied on the right side, over the external orbitar process, reaching to the coronal suture. The long-continued and repeated efforts which were persevered in before the cranium could be moved, were extraordinary. After two hours' hard application, the head was forced down. The child had been dead for many hours. The vectis had borne in that side of the frontal bone upon which it was fixed.—The placenta was soon afterwards found detached and extra uterine, and was easily extracted. It required some care to prevent protrusion of the intestines along with the placenta. There was no farther flooding. The patient still complained of great pain and tenderness. She was much exhausted ; and neither her general appearance nor pulse augured favourably of the termination of her great sufferings. The difficulty of breathing was of course much relieved. Opiates were given at intervals throughout the next four-and-twenty hours, and some subsidence of the vomiting and violent pain were procured. On the following morning, more to my surprise than otherwise, I found the severity of the symptoms, generally, ameliorated. The vomiting had almost ceased; the tenderness of the abdomen had not increased ; still I thought it a safe precaution to apply twenty leeches, and keep up fomentations. Saline mixture with laudanum was administered every four or six hours. On the 9th, the countenance began to lose much of its anxiety, and the tenderness and pain to decrease. The bowels were readily relieved by a small dose of castor-oil, and the urine was voided with ease. The pulse was also much improved, being considerably less frequent, and softer. Though we naturally calculated on some injury to the soft parts, from the strong application of the vectis, I was agreeably surprised to find that no sloughing, or other inconvenience had taken place. On the 18th day she was able to sit up, and by the 30th was able to resume her journey.—*Lond. Med. Gaz.*

3. *Alteration of the Blood in Yellow-Fever.*—At a meeting of the College of Physicians, a paper was lately read, entitled "Observations on the Blood," by Dr. Stephens. The object of the author was to prove that the proximate cause of yellow fever was to be found in the changes which the blood undergoes, and that the true remedy for this and other fevers, consists in opposing by proper means those changes. * * * * *

"On opening the heart," says Dr. Stephens, "we find, instead of blood, a dissolved fluid, nearly as thin as water, and almost as black as ink. In both the cavities of the heart the fluid is equally black, and in the whole vascular system, all distinction between arterial and venous blood is entirely lost. In this state it is quite evident that the blood is incapable of stimulating the heart or supporting life." In the progress of the fever, the changes of the blood succeed each other in the following order:—1st. The blood loses its solid parts, and becomes thin. 2d. It loses its saline principle, and becomes black and vapid. 3d. Its preservative elements are now dissipated, and it goes fast to decay, the more rapidly, says Dr. Clanny, as no new blood can now be formed. 4th. It loses its vitality, and is incapable of supporting life.

* * * The treatment consists in allaying the excitement, if any exist, during the first twenty-four hours, by venesection, mild purgatives, and sponging the body with cold-water; the saline medicines, which are considered the best agents for preventing the decomposition of the blood, are then exhibited in small and repeated doses. Those usually preferred are the Rochelle salts, and the carbonate of soda, potass, and ammonia, &c. This practice was tried in the hospital at Trinidad, in 1828, a season peculiarly sickly in that island.—It was applied in three hundred and forty cases, including the remitting and yellow fevers, admitted into hospital, after the symptoms had existed variously from six to seventy-two hours, with such success, that not a single case proved fatal. Dr. Stephens says it can be clearly proved, in the West India fevers, that those patients that are left to nature have a better chance of recovery than those who are treated with emetics, calomel, or antimony, opium, or acids ; and that these remedies decidedly increase the very evils they are meant to relieve, and add greatly to the mortality of fever in hot climates. Dr. Clanny maintains the same opinion as to their effects in the typhus of cold climates. Dr. Stephens states, that those who attend only the solids and the mere excitement, can never cure a single case of yellow fever that is really severe. From the results of this plan of treatment, not only in their hands, but in those of Dr. Stedman, of St. Thomas's, and Mr. Greatrex, of Trinidad, it must be, as it deserves, generally adopted. Whilst these fevers were considered as diseases of the solids only, and treated as such, no efficient control could really be exercised over them. This is a proposition the most determined localist cannot controvert. Wherever this treatment has been tried, its authors assure us that these hitherto terrible scourges have been disarmed of their terrors.

4. *Dr. Middlemore on Amaurosis.*—The following remarks are chiefly on the exhibition of strychnine in that formidable malady, amaurosis. It is a remedy which is now in some vogue, not only with oculists, but with physicians also, in the palsy-cases of middle and advanced age, whether the affection be of the retina or of one side of the body, of a solitary muscle or a large portion of the material frame. The very nature of these cases—we mean their connexion with advanced life, and their dependence on the wear and tear of the machine—precludes the hope of any remedy or class of remedies proving extensively and permanently useful. Medea's cauldron would suit such cases, but short of the youth-restoring drug of the enchantress, there is nothing, alas! that will give elasticity to the withered sinew,—plumpness and vigor to the wasted muscle,—or the finely-tuned sensibilities of adolescence to the palsied nerve. Nevertheless, there are, undoubtedly, some cases of palsy and amaurosis—we fear they are fewer than is imagined—that are benefitted by the exhibition of the strychnine, or stimulating medicines of that class. The discrimination of these must be a matter of experiment and experience. We will give the results of Mr. Middlemore's.

"If a patient has overworked the eye by a long-continued action, confined to the inspection of objects of the same color and description, an enfeebled condition of retina (just as we produce an exhausted state of muscle by over-exertion) will take place. If a man subject his eye to an unnatural stimulus, by looking for many hours daily at bright substances of the same or nearly the same color,—or to sudden transition from an artificial glare to comparative darkness (as miners)—or to a diminished stimulus, as by working in dark rooms, or places imperfectly supplied with light,—or to any cause allowing the visual textures of the eye to remain for a long period in a state of inactivity, as takes place where large opacities of the cornea, and fully formed cataract exists,—the power of the retina will be partially destroyed; its susceptibility to the stimulus of light diminished; but in none of these cases will there be found any structural change in the retina or the optic nerve, any congestion of vessels, or any discoverable alteration from a healthy and natural condition: nor will the system, in all probability, be found affected; no altered state of health sufficient to account for the dimness of vision, will be found to exist. At some kinds of employment it is necessary for the individual to work with the head bent forwards, declining, or the body so distorted as to favor the too liberal flow to the eye, and retard its return,—inducing what is termed congestion: a distended state of vessels, unfavorable to free and active circulation; a condition of eye which is also frequently induced by the investigation of minute objects by the aid of powerful glasses. Loss or diminution of the power of vision sometimes comes on from certain causes which diminish the vigor of the constitution generally—as, for instance, after profuse salivation, long-continued suckling, menorrhagia, &c. In all these cases, I believe, the strychnine is calculated to produce great and permanent advantage, in combination, of course, with other remedies suited to the particular exigences of the case,—for example, if the retina be weakened in consequence of diminished vigor of the system, remedies adapted to strengthen the system, and a removal of the cause enfeebling it, might be joined to the local application of the remedy in question. But the power of the retina will not always return with the returning strength of the system;—in such cases the strychnine is singularly valuable, producing, with wonderful rapidity, the restoration of the organ of vision. Strychnine, given internally, does not produce the same beneficial effect as when applied externally. The mode of using it is already before the profession. After having tried it in a variety of ways, and in different situations, I have not been able to discover a better method than that of blistering the skin above the eye-brow, and, after having carefully removed the cuticle, I sprinkle the powder upon the raw surface, taking care to pass a spatula upon the part so sprinkled, to secure it against removal, and ensure its absorption: a piece of lint, not greased, should afterwards be bound upon the part. The quantity with which I generally commence, is the twelfth of a grain upon each side, daily augmenting the quantity, as the patient is able to bear it, to two thirds of a grain upon each blistered surface. Its first effects are—slight pain in the head, increased power of vision, and severe smarting pain of the part upon which it is applied. Some patients cannot bear its application; others require great care, and a very gradual augmentation of the quantity to enable them to bear it; whilst others will admit of its application without experiencing any other uneasiness than what arises from its action upon the sore. It is not necessary I presume, to detail cases in support of my views; such a plan would greatly extend my observations, which I have been studiously anxious to limit.

I will now, for a short time, draw the attention of your readers to those cases in which the employment of this remedy would be useless or injurious. If the amaurosis be dependent on any morbid condition of the brain; any alteration of the bony structure; any tumor or other substance pressing upon the optic nerve, the effects of former inflammation, such as opaque deposition or partial disorganization, the effusion of blood or morbid growths, the enlargement of the vitreous or displacement of the crystalline humor, producing pressure upon the retina; a varicose state of vessels, as a consequence of distention so continued as to impair their tonic and elastic properties; inflammation of, or disease of, those parts encased by, or anterior to, the retina,—no benefit could be expected to result from the use of strychnine; but, on the contrary, in many of the cases, material injury might succeed its employment."—*Medico-Chirurg. Review.*

5. *Gonorrhœa alternating with Bilious Remittent Fever.*—By Lewis Campbell, M. D., of Moutton, Alabama.—On the 23d of July, 1827, I was called by Mr. ———, who had been affected with gonorrhœa three years since; and as he had been obliged to ride some distance every day on business, the symptoms were somewhat aggravated. He suffered greatly from ardor urinæ and chordee. The discharge of matter from the urethra was very profuse, and considerable general excitement was present. His bowels were freely opened with sulphas magnesiæ; one drachm of balsam of copaiva was directed to be given three times a day, and a low diet enjoined. He obtained sudden relief of the ardor urinæ from this course; but a profuse discharge of matter, and the retraction of the urethra, remained. His business calling him from home, he was under the necessity of riding some distance every day till the 26th, when he was attacked with a bilious remittent fever. During the evening he felt languid, sick at the stomach, &c. At eight in the evening he was attacked with a violent chill, followed by fever. During the chill the gonorrhœal discharge was suddenly suppressed. On the morning of the 27th I found him entirely free from all the symptoms of gonorrhœa. He assured me he felt no inconvenience from that source.

The fever continued till the 30th. During this time he enjoyed entire exemption from the gonorrhœal symptoms. On the 30th, his general health being much improved since the preceding day, he escaped fever, but was again attacked with gonorrhœa and chordee. He was again put under treatment for those affections. His general health continued to improve till the 2d of August, when from exposing himself to the sun on a short ride in the heat of the day, the bilious symptoms returned; and he then had chills and fever two days in succession. During the first chill the running was again suppressed; and he was completely relieved from any uneasiness in the urethra during the continuance of the febrile symptoms. On the 14th he experienced exemption from fever and a return of gonorrhœa. His general health continued to improve from this period, though the affections of the urethra proved somewhat lingering. His stomach rejected the balsam in any form, and scarcely tolerated the presence of any medicinal substance whatever. Regimen and time effected a cure. Was the suppression of the gonorrhœa during the fever, and its immediate return when the fever yielded, a mere coincidence? Or was it a metastasis of morbid action from the urethra to the stomach, and from the stomach to the ur thra? If the latter, does it not favor the opinion of the physiological school relative to the non-specific nature of venereal affections?—*Bost. Med. and Surg. Jour.*

6. *Mysterious Expectoration of Pus.*—A case related to the Medical Institutions of Paris by M. Merat occasioned a very warm discussion among the members, and, as usual, drew forth many curious facts and observations. The original case was as follows :—A young lady expectorated twice a day, for a long time, a large quantity of purulent matter, of a very fetid odor. The expectoration occurred early in the morning, and again at two o'clock p. m. The paroxysm, which generally lasted only a few minutes, was preceded by a sense of suffocation—then came on a slight cough, and a discharge of a tumblerful of pus in a few minutes. The chest was examined with the stethoscope, and it was thought that an excavation existed in the posterior and lower portion of the left lung, but they do not appear to have been very positive as to that point. This young lady had been subject to attacks of this kind for some years, and married, contrary to the advice of M. Merat, at an early age, and became pregnant. She got to the eighth month of uterior-gestation, having frequently required venesection. The expectoration of purulent matter ceased, and this lady died in two or three days' illness. On examination, (which was unavoidably confined to the thorax) no trace of disease could be found in either the lungs or their coverings. There were no adhesions—no trace, in short, of any malady whatever.

M. Gendrin remarked that, as hepatic abscesses sometimes make their way through the diaphragm, and the matter is evacuated by the trachea, this might be a case of the kind. It is astonishing that, in such a society, no one observed that such a thing was impossible in the present case, where there were no adhesions, nor any breach of structure in the diaphragm, through which the hepatic pus might pass. M. G. related the case of a lady who had suffered for several years from attacks of puruleut expectoration, preceded always by engorgement and tumefaction of the right hypochondrium. The matter was ejected by a combination of vomiting and coughing, after which the region of the liver diminished for two or three weeks, when the symptoms were renewed. This female is still living, and has been seen by Recamier, Dubois, and many other eminent physicians of Paris.

M. Sandras conceived it probable that, in M. Merat's case, the puriform matter came from the stomach—the cough and sense of oppression preceding and accompanying the discharge being no positive proof that the matter came from the lungs. M. Merat himself acknowledged that he now came to a similar conclusion, in which, indeed, we entirely agree. M. Merat declared, however, that the pus on repeated examinations, was pure, and without the least admixture, apparently, of mucus. Various opinions, supported by cases, were brought forward respecting hepatic abscesses, but these we need not detail.—*Ibid.*

7. *Experiments upon the Coloring of Different Tissues.*—M. Casimer Broussais, at a recent *concours*, had given to him, as the subject for his thesis, the following question. Can we distinguish by certain appearances in the dead body, the organic alterations which have commenced simultaneously with the disease, and those which are formed during its course, at the moment of, and after, death? In order to be enabled to answer this very important question, M. B. instituted a series of experiments, the conclusions derived from which we shall lay before our readers.

To treat this question clearly, usefully, and without repetition, M. B. thinks it convenient to divide the different lesions of the body into five species, as follows: alterations depending upon, 1st, acute inflammation; 2d, subacute or chronic inflammation; 3d, atony; 4th, physical causes during life; 5th, the dissolution of the organs and the fluids after death. The following are the principal results of his experiments.

1st. The tissues that are perfectly white and completely exempt from inflammation, do not become red on exposure to the air, as Professor Laennec has asserted.

2d. The white and healthy tissues redden on maceration in blood, some with greater and others with less facility; but it is to be remarked that this red colour soon becomes deep brown, and livid, if the maceration be continued beyond a certain time; moreover, the diaphanous pellicle which forms the exterior covering of the serous membranes, and similar to that which forms the internal surface of the arteries and veins, also becomes coloured ; finally, if the maceration be continued longer than one day, the tissue commences to be friable. But if the tissues reddened by maceration in blood be placed in pure water, at the end of twenty four hours, there will not remain any trace of the colour.

3d. The red and inflamed tissues suffer on maceration in pure water the following changes ; if they have a very slight redness, this disappears after maceration, and reappears on exposure of the tissue to the air ; but if the redness be very bright and deep, it loses only a little of its intensity. Membranes with several spots of chronic inflammation, around which recent inflammation was developed, exhibited phenomena the most interesting and confirmatory of the principles of my father in relation to circumscribed local phlegmasiæ.

4th. In the serous and sanguineous vessels, the diaphanous membrane of which we have spoken, does not become red from inflammation, at least unless the irritating cause acts upon it directly, or unless in the progress of the disease, the redness has passed from the subjacent tissue to it, which is of very rare and difficult occurrence.

5th. The redness caused by imbibition, from maceration, is not rose-coloured, except at first ; it soon becomes of a deep shade and livid ; it is always uniform, and never in points or aborescent ; finally, it readily and completely disappears on maceration in pure water.

6th. The redness from inflammation is sometimes aborescent and in points ; but it is often also uniform, as the injection of ammonia into the carotid of a dog has proved. In this last case it may be deep coloured and black, but not livid, as when coloured by imbibition, and when it does appear similar to this last, it may be easily distinguished, since it does not disappear by maceration in pure water. If, on the contrary, it be light and rose-coloured, it then has a liveliness of colour which belongs only to it ; moreover, although it disappears by washing like slight colouring from imbibition, it does so less readily ; finally, what is a curious fact, if after this light redness has been made to disappear by washing, the tissue be exposed to the air, the colour soon reappears, which never takes place in tissues coloured by imbibition of blood.—*Annales de la Medicine Physiologique, January*, 1830.

8. *Hernia of the Stomach, with Enormous Enlargement of that Organ*.—This case was communicated to the Royal Academy of Medicine by M. Yvan. The subject of it was a soldier, who for some years had had a scrotal hernia, which could be easily reduced by taxis, but could not be kept up ; it gradually increased in size, and a month previously to his death repeated vomiting took place, which could not be arrested, although there was no strangulation, and the patient died. On examination, the abdominal ring was found to be enormously distended, measuring eighteen inches in circumference; the hernial sac contained the inferior third of the stomach, the greater omentum, the small intestines, and the large intestine, except the iliac portion of the colon, the cœcum, and the rectum. The stomach, situated parallel to the axis of the body, was of an enormous volume, and seemed divided by a circular depression into two portions! the one situated in the abdomen, the other in the hernial sac. The length of the great curvature of the stomach was three feet, that of the lesser curvaturé eighteen inches; its circumference at its largest part twenty inches, and contained nearly eleven pints, (*five litres,*) of fluid. Longitudinal and circular muscular fibres were observed on the surface of this organ. M. Y. deposited the stomach in the Museum of the Academy.—*Archives Generales, Jan.* 1830, *and Revue Medicale, Feb.* 1830.

9. *Phlegmasia Dolens in a Male.*—A. Copland Hutchinson, describes in the 15th Vol. of the Transactions of the Medico-Chirurgical Society of London, the following case:

Mr. B. received a blow upon his right shin, about twelve months ago, immediately over a branch of the sphena vein, by a small piece of timber accidentally falling upon it. The scar is very slight, though the injury and its results appear tó have been severe, and the patient states that the accident was followed by considerable swelling and inflammation all over the limb, and that the abraded surface was very long in healing. Mr. B. says he first felt pain in the direction of the upper third of the saphena before it actually dips to unite with the femoral vein. The whole leg and thigh soon beeame enlarged and inflamed, the former partly œdematous; and although the patient states the disease to be slowly on the decline, yet the enlargement of the thigh and leg still continues, and he has pain from the groin to the heel and sole of the foot, principally in the direction of the branches of the saphena, with a slight blush of redness over the forepart of the leg, where the original injury was received; but while the member is kept in the horizontal position he is nearly free from pain.

I have traced the upper portion of the saphena vain, aud find it to be a complete ligamentous cord for eight or ten inches, but the femoral vein seems to me to have escaped thc diseased action. The patient has no pain or uneasiness within the pelvis, and his general health is good. It should be stated, however, that the testis of that side is slightly enlarged, but not indurated.

Dr. G. R. Treviranus also relates in the 5th Vol. of the Heidelberg, Klinische Annalen two cases of this disease occurring in men. Dr. T. considers the disease to be a catarrhal affection of the cellular tissue surrounding the muscles, and of the mucous sheaths.

10. *On the Morbid Phenomena caused by the use of Iodine.* By Dr. Jahn.—"The author of this paper, published in the Journal Complem, for Feb. 1830, informs us that in the part of the country where he resides, there prevails a species of bronchocele which, to all appearance, is a simple hypertrophy of the thyroid gland. He affirms that this affection is never seen where the water springs from ground that is basaltic, porphyritic, granitic, or gravelly: whereas it is constantly observed where the waters issue from calcareous soils, and contain a quantity of lime in mixture. Iodine is the remedy which is generally employed for bronchocele in this part of the country, and he has had ample opportunities, he says, for remarking its effects on the animal organization. Orfila showed that this powerful medicine, in large doses, occasioned pains in the stomach, malaise, vomiting, diarrhœa, syncope, oppression, ptyalism, tremors, &c. Symptoms of this kind were observed by our author in a man who had swallowed, by mistake, an inordinate dose of the tincture of iodine. In other cases where the medicine was merely continued longer than proper, Dr. J. was struck with the slow but great absorption of fat, preceded and accompanied by an augmentation of all the secretions and excretions. He observed the skin became dark colored, and the perspiration viscid—the breathing embarrassed—the urine abundant—the bowels loose and very bilious—the catamenia (in females) more copious. From these phenomena our author argues that the functions of the venous and absorbent vessels are much increased, in proportion as the nutritive system has been diminished. The blood, he says, becomes thinner than natural, and less abundant in fibrin. If the medicine be continued in these cases, various bad consequences ensue. He mentions two cases where he opened the bodies of patients who labored under the excessive use of iodine, and where the emaciation was extreme, and the viscera in a very flabby state. Still Dr. J. considers the medicine in question as one of the most valuable which has been discovered within the last hundred years. Indeed he terms it 'un remede divin.'

"In scirrhus of the pylorus Dr. J. affirms that iodine has sometimes removed the malady, when early applied, and when aided by leeches. The same has been stated by Wagner, Henneman, Hufeland, and others. We would strongly recommend the medicine in question to be tried in dropsical cases dependent on visceral obstructions."—*Med. Chirurg. Rev. April*, 1830.

11. *Clinical Reports of the effects of Venesection in the cold stage of Intermittent Fever.*—This report was made by Mr. Twining to the Medical and Physical Society of Calcutta, at their meeting in Dec. last, and comprehends ten cases.

Case 1. Was bled to twelve ounces in the cold stage of the 6th paroxysm of tertian intermittent. He experienced immediate relief, the rigors ceased, and he became hot for about half an hour; he had a slight return of fever daily at noon for six days, not preceded by rigor or cold. This patient had enlarged spleen.

Case 2. Was bled to fourteen ounces in the cold stage of tertian ague in the 4th paroxysm. The rigors soon ceased, he had a slight hot stage for about half an hour, and there was no return of the disease.

Case 3. Was treated with purgatives, quinine, and arsenic, afterwards with mercury to salivation, without benefit; bled in the cold stage of the 11th paroxysm, he felt immediate relief, and had a very short and slight paroxysm without sweating stage. A slight feverish feel remained for eight days after. At the end of fourteen days, he had a return of ague, and on the return of the next paroxysm, after one day's interval, he was bled in the cold stage, and cured.

Case 4. Quotidian ague of seven day's duration, purged and took bark, bled to eighteen ounces in the cold stage of 7th paroxysm with great relief; was exposed to cold next night, and had continued fever afterwards.

Case 5. Tertian ague, bled to lbi. in the cold stage of the 5th paroxysm, with immediate relief; had a short and slight paroxysm, and was cured.

Case 6. A most distressing tertian, with very severe rigors; bled lbi. in the cold stage, and cured.

Case 7. Irregular ague, sometimes tertian, sometimes quotidian, bled to twelve ounces in the cold stage, with much benefit, he was cured, having in place of ague on the two next days of expected access, a slight feverishness.

Case 8. An Asiatic, a native of Madras, bled to six ounces and a half in the cold stage of 2d paroxysm of tertian. The rigours ceased in less than two minutes after the vein was opened, he had no fever, and was cured.

Case 9. Irregular intermittent, used quinine without benefit, emaciated, bled to six ounces, in cold stage, with immediate benefit, and had no return of the disease.

Case 10. Bled to lbi. in the cold stage of 6th paroxysm, the cold ceased, and he had a slight paroxysm. The ague returned on its regular day, and he was bled again to ten ounces, in the cold stage, which was arrested and had no ague or fever since.

Mr. T. enters on some remarks as to the benefit to be expected from guarding against the ulterior results of repeated congestion of internal organs. He also observes, that further experience is requisite to prove whether the treatment be adapted to all cases of ague, at all seasons, and in all situations, or to persons debilitated by disease and long residence in India. Mr. Twining usually has dr. ss. of sp. ammon. aromat. mixed with oz. i. ss. of warm water ready, in case the patient be faint; he says this is rarely used; but that the patients appear better for drinking a cup of hot sago or tea within half an hour after the bleeding.

12. *On the Treatment of certain Injuries of the Eyes.* By Samuel Barton, Surgeon to the Manchester Eye Infirmary.—In cases in which the cornea has been punctured by a sharp body, and the capsule of the lens wounded at the same time; and also in those cases in which, without any penetrating wound of the cornea, the capsule has been ruptured, and the lens forced into the anterior chamber, Mr. Barton recommends the early extraction of the lens, as the means of affording the patient the best chance of escaping with the least injury of vision and deformity. The usual modes of treating such accidents is generally to subdue inflammation, by venesection, leeching, &c. and waiting for the absorption of the lens. This practice, though admitted by Mr. B. to be sometimes successful, he says in a majority of cases we shall have to contend with a tedious and severe inflammation in the first instance, and eventually an obliterated pupil, with a thickened capsule, adhering firmly to the iris, that cannot be dislodged, and not unfrequently suppuration of the eyeball.

"My attention was directed to the improved treatment of these cases of early extraction of the lens," says Mr. B. "from comparing the advantages with the disadvantages, resulting from the different operations for cataract. From observation I found that more inflammation of the eye, and more constitutional disturbance, ensued from the operations for the depression of the lens, and for its solution, than from extraction, whenever the last operation was well performed.

"It will be an objection with some surgeons to interfere with a single cataract, when the vision of one eye is perfectly good, and in most cases of spontaneous cataract I should agree in this opinion; but in the accidental cataract I should, from experience, recommend the operation of extracting the lens without delay. My mode of performing the operation varies very little from that of the usual manner of extraction; but in making the incision in the cornea, I am governed with respect to the extent of it by the state of consistence of the lens, and the situation of the wound of the cornea.

"As in young subjects, the lens will be generally soft, if the situation of the original wound of the cornea produced by the accident be not unfavorable, a smaller division of the cornea will answer the purpose than when the lens is hard. When the accident takes place in an old person, we have reason to expect to meet with a harder lens, consequently a larger division of the cornea is required, provided that the nature of the case will admit of it.

"In performing the operation, I generally use Beer's extracting knife, and having carried the point into the centre of the pupil, I then raise the handle of the instrument so as to depress the point, and keep it there for a few moments, until I have ascertained whether the lens will escape, by merely making a slight pressure with the knife kept in this position—and in many instances this takes place. If I find that the lens is too hard to pass, I extend the incision before I withdraw the knife, and finish the operation with the scoop.

"Although I am an advocate for extracting the lens early in such cases, there are certain injuries of the cornea in which it will be necessary to wait until some reparation has been made: for instance, when the cornea has been divided by any rough cutting instrument, producing an angular wound with ragged edges, it will be desirable to wait and employ those measures which are most likely to restore union.

"After my early operations, I dressed my patients either with a wet cloth, or a plaster of simple cerate, and a bandage placed lightly over the eye. By such loose dressings it is scarcely possible to keep the eye in a sufficiently quiet state till perfect union of the cornea shall have taken place ; and it was evident to me, that I had not only very great difficulties to encounter in the after-treatment, but that the operation of extraction failed in some cases in consequence of such dressings. I therefore determined to employ those means that were the most likely to keep the eyelids steady, without producing pressure, and thereby promote the union of the wound of the cornea by the first intention.

"After the pupil has been cleared of the lens, the eye is to be treated as in cases of extraction ; and the plan that has proved the most successful in my practice is to place two slips of court-plaster, four inches in length, and a third of an inch broad, in a perpendicular direction across the eyelids of both eyes.

"This I find to be the best dressing, since without occasioning pressure or uneasiness, it at the same time allows the discharge of any fluid.

"If the process of healing be not interrupted, we shall find in the course of three or four days that all discharge has subsided. The patient will sometimes complain of a pricking sensation in the lids, and it will then be necessary to remove the plasters carefully, and replace them by new slips ; but in changing the plasters, the eye should not be open for an examination until the fifth or sixth day after the operation.

"Since I had recourse to this dressing after the operation of extraction, my cases have been very successful, and attended with less trouble and anxiety, than when I have operated by depression or solution : and I can confidently recommend it when either the cornea has been opened to make an artificial pupil, or in injuries from accidental circumstances ; and the same applications of the slips of court-plaster will be found advantageous, by those who prefer the operations for depression or solution."—*London Medical Gazette, March*, 1830.

13. *Amaurosis.*—A young female was, at the beginning of November last, brought to the Hotel Dieu on account of a complete amaurosis after a blow on the right eye with a whip. No wound could be discovered ; the conjunctiva was slightly ecchymosed, the pupil dilated and insensible, and vision completely destroyed. The humours of the eye were perfectly transparent. She was bled, and had leeches applied to the temples, but without any immediate benefit. On the third day, however, she began to distinguish light, and after a few days more, vision was almost completely restored.

Cases of Amaurosis, M. Dupuytren observed, after contusions of the eye, are by no means unfrequent ; but it is worthy of remark, that this effect should sometimes be met with in cases where the external violence appears hardly sufficient to produce such an effect. Thus, corks of champaigne bottles, striking against the eye, have, in some instances, produced amaurosis, and a similar cause exists, perhaps, in all cases of so-called spontaneous amaurosis, which takes place suddenly. M. Dupuytren mentioned another case of amaurosis after a blow with a whip, where there was apparently no wound, but considerable ecchymosis, and swelling of the conjunctiva; violent inflammation succeeded, and in spite of the most active antiphlogistic treatment, terminated in the evacuation of the humours, in the midst of which one of the knots of the whip was discovered.

14. *Portion of Bone lodged for forty-eight days in the Trachea of an Infant.*—By Thomas Stabb, M. R. C. S. &c. On the 20th of Sept. last, S. H. S., aged ten months, playing with a bone of a neck of mutton, which the nurse gave her whilst at dinner, put it into her mouth and detached a small portion, about the size of a large marrow-fat pea, which slipped into her windpipe, and produced violent coughing and irritation for about five minutes, when it ceased, leaving a noise in breathing like that produced by a saw. In the course of twenty-four hours, great difficulty of breathing, with constitutional irritation and cough, came on, which was subdued by the usual remedies. The same saw-like noise of breathing, and some cough, continued, but did not appear to give pain after the fourth day, the child's health and spirits after that time appearing as good as usual, except this constant wheezing.

On the 3d of November, upwards of six weeks after the accident, in consequence of a cold she took from going out into the air, violent irritation in the trachea, with cough, returned. A solution of tartarized antimony was given, and on the 7th, after a dose which produced vomiting and general relaxation, and whilst the nurse was briskly rubbing her throat with a volatile embrocation, the head being bent back over her lap, she was seized with a violent fit of coughing, and threw up the piece of bone, embedded in mucus, which had been retained forty-eight days in the trachea. Her breathing almost immediately became natural, and the next day she was as well as ever. The piece of bone was very rough, of a triangular shape, the edges quite sharp.—*Lond. Med. Gaz.*

15. *Strabismus.*—Dr. Rossi, of Turin, thinks that this defect of vision, when it is congenital, is owing to the form of the orbit, the axis of which, instead of being perpendicular to the face, forms with it an acute angle. He accounts, upon this theory, for the fact, that congenital strabismus is often spontaneously cured as the parts increase by growth and acquire new proportions. Dr. R. has invented a kind of glasses which he thinks calculated to remedy this disease, when arising from other causes than the one just alluded to. The glass intended for the affected eye is rendered opaque by covering it with a black pigment. Two orifices are then made through it, one perpendicular to its surfaces, and the other crossing and bisecting the first, but at such an angle that its direction is parallel to the usual axis of vision of the distorted eye. The second perforation is not cylindrical, but diminishing towards the eye, and enlarging externally. The glasses are, of course, plane. The effect of this arrangement is readily conceived. The direct perforation affords a sufficient means to the eye of seeing objects in front, so that the utility of the organ is not diminished. The other forms a tube which, enlarging and becoming lighter towards its external part, attracts the notice of the eye, the axis of which is thus directed in a line the reverse of that of its usual diseased vision. The effect thus produced is continued until the morbid habit is overcome, and the eye is enabled to resume its appropriate functions.—*Bost. Med. and Surg. Jour.*

16. *Protracted Utero-Gestation.*—Unequivocal proofs exist that the period of gestation has been prolonged for several weeks beyond the period usually assigned to it. From the many cases which have been adduced in evidence of this, we select the two following, which are not so remarkable for the amount of time by which the period was exceeded, as for having been accompanied by circumstances which determined exactly the time of impregnation. The cases are taken from the minutes of a meeting of the Westminster Medical Society, reported in the London Medical Gazette. The first occurred to Professor Desormeaux, of the University of Paris, who states it as follows:—

"A lady, the mother of three children, became insane. Her physician considered that childbearing might have a beneficial influence on the mental disease, and permitted the husband to visit her, but under condition that it should be only once, and at the distance of three months, in order that, if conception took place, there might not be a chance of abortion, from the circumstance of any further intercourse. The physician and attendants made an exact note of the time when the husband was permitted to visit his lady. When, at last, symptoms of pregnancy appeared, the visits were absolutely and totally discontinued. The patient was necessarily watched by the female attendants required for her malady, and was, moreover, a lady of the strictest principles of morality. She was delivered, at the termination of nine solar months and a fortnight, of a *small* child, and Professer Desormeaux delivered her."

The other case, which is related by Dr. Dewees, is as follows:—

"The husband of a lady was obliged to absent himself from her, in in consequence of embarrassment in his affairs. He returned one night clandestinely, and his solitary visit was only known to his wife, her mother, and Dr. Dewees. The consequence of this visit was conception, and the lady was delivered of a healthy child in nine months and thirteen days after that nocturnal visit.—*Ib.*

17. *Poisoning by Opium and Belladonna, used as an Injection.*—Madame * * * *, æt. 22, had been tormented for some months by a dartrous eruption on the vulva. Having tried various means without relief, she applied a strong solution of opium and belladonna, which gave her some ease. On the 19th of November, 1829, she determined to use the narcotics in the form of enemata, and mixed a pint of the lotion with sufficient water to make three injections, each containing a scruple of opium and half an ounce of the leaves of belladonna. She took the first at eight o'clock in the evening, and the second and third shortly after the first; all three were returned, and some feces were discharged. At half-past eight she went to bed, feeling rather confused. Her husband, a medical man, found her sleeping soundly at half-past nine, and did not disturb her. At midnight, however, finding her still buried in the deepest slumber, he became alarmed, and sent for Dr. Solon. Her face was now extremely pale, the pupils dilated to the utmost, the tongue dry, deglutition difficult, the respiration short and frequent, the pulse small and 130. The limbs were perfectly motionless, and the skin insensible to pinching or irritation of any kind. A purgative injection was immediately exhibited, a free bleeding practised from the arm, ten leeches placed behind each ear, sinapisms applied to the thighs and legs, and ether draughts administered by the mouth.—At 5 o'clock next morning the patient opened her eyes, uttered a few unconnected words, and vomited some bilious matters. In the course of an hour she awoke, as from a painful dream, recollecting nothing of what had occurred. Vision was imperfect; she appeared to see things through a thick veil, and whenever she closed her eyes her ideas became confused and incoherent. We need not pursue the details; suffice it to say that the lady recovered from all her severe symptoms. The parts, however, to which the sinapisms had been applied, sloughed, in consequence of the length of time they had been continued, and many months elapsed before she could walk about. The eruption on the vulva was quite cured by this severe discipline, and the patient subsequently went well through a confinement.—*Medico-Chirurg. Rev.*

18. *Notice of Iron found in the powder of Cinchona bark.*—By Charles Ellis.—A circumstance occurred with a highly respectable house in Baltimore, to which we are indebted for a knowledge of an accidental impurity in the powder of cinchona, which is believed to be of sufficient importance to interest the readers of the Journal.

An ounce of calisaya bark *in powder*, was procured of them, and directed by the physician to be made into a decoction. The liquid when decanted was nearly the color of ink. A second ounce was obtained and infused in an earthen vessel, with precisely the same result. The conclusion was, either that the bark or the water contained iron; and to determine to which of these causes to assign this change of color, and to ascertain whether the bark were really impure, these gentlemen submitted it to the following experiments, viz:—1. A small quantity of the powdered bark was examined by the aid of a microscope, and the whole surface found studded with small metallic specks, some black, some bright, giving it quite a lustre. 2. A quantity of the powder was boiled in a Florence flask with distilled water. The decoction was of a deep black color, taste similar to ink, and entirely devoid of the sensible properties of a decoction of pure cinchona; suffered to stand, the supernatant liquor was of a greyish blue color, and the precipitate of a dark brown, approaching to black. 3. A quantity of the powder was exposed, in a shallow vessel, to a stream of water, so as to wash away the lighter particles, and the deposit left in the bottom of the vessel consisted of small black grains of a metallic lustre. The inferences drawn from these experiments were, that the powder contained a metallic substance which proved to be iron; and that the precipitate in the decoction was owing to the action of the components of cinchona upon the iron.

These results led to an examination of other parcels of powdered bark, and in upwards of twenty different samples examined by my friend John Farr, and a number by myself, there were none in which the magnet did not detect minute particles of iron; in some much fewer than in others.

In order to ascertain the amount of impurity in a given quantity of bark, I washed carefully half an ounce of the same lot used in Baltimore, and obtained one grain of iron in a metallic state: there was perhaps from a fourth to half a grain lost in the operation. From one ounce of another parcel there was half a grain separated, by the magnet.

It will be readily perceived, from the nature of this admixture, that it was entirely accidental, and fortunately not of a character calculated to do any injury.

Inquiry having been made of the powderer, it was ascertained that his machinery does not materially differ from that in general use; that the revolving stone is shod with iron, and passes over a cast-iron plate; a sufficient cause for the existence of minute particles of iron in the powder, particularly as in this instance the bark was not dusted, a process by which the impalpable powder is separated from the heavier and coarser particles.

Although it is not probable that the quantity of iron found in this cinchona would render it objectionable in many cases, still it is desirable at all times to have our remedies free from all foreign admixture; that the physician may know precisely what he is directing, and the patients may neither be alarmed nor disgusted with unexpected, and to them unaccountable dangers. From the well-known hardness of the French burr stones, we may readily conclude that bark might be ground by them without the fear of adulteration.

It may be observed, in passing, that barks, roots, &c., of nearly every kind, are more eligible for decoction or infusion when coarsely powdered, or bruised, as it is technically called, than when reduced to an impalpable powder.—*Phil. Jour. of Pharm.*

19. *On aromatic or spiced Syrup of Rhubarb.*—By Elias Durand.—Dr. Coxe, in the last edition (1830) of his American Dispensatory, has very judiciously observed that this syrup, prepared agreeably to the Pharmacopœia of the United States possesses a defect which may be easily obviated, without changing the proportions of its ingredients. In fact, evaporating to one half an infusion of rhubarb and aromatic substances, is quite inconsistent with the present improvements in pharmaceutical manipulation; it is too well known that these articles lose, by ebullition, a great portion of their active properties.

This fault, as well as many others which have crept into that national work, has not escaped the attention of our practical pharmaceutists. From the first time I had to compound the aromatic syrup of rhubarb, this defect struck me, and I amended the formula by the following, which undoubtedly affords a preparation very superior to the other, both in nicety and activity. I first prepare an alcoholic tincture with the rhubarb and the aromatic ingredients, and then form my syrup by the addition of a relative quantity of simple syrup.

*Aromatic Tincture of Rhubarb.*

R. Rhubarb of good quality, parts v.
Cloves and cinnamon, of each, parts iv.
Nutmegs, part i.
Alcohol of 20 deg., parts lxiv.

Bruise the ingredients and macerate them for about a week.

*Aromatic Syrup of Rhubarb.*

R. Aromatic tincture of rhubarb, part i.
Simple syrup of 35 deg., parts iii.

Mix well. This syrup marks 28 deg. on Baume's pese syrup.—*Ib.*

20. *Formula.*—In foreign pharmacopœiæ and other works of pharmacy, we often find formula for combinations, that are entirely unknown to those of our own country. We insert the following from Verey's Pharmacy, without any respect to arrangement.

*Silk Plaster Cloth.*

R. Isinglass 1 oz. 1 dr.
*a* Alcohol 22 deg. Baume 12 oz.
Tincture of Benzoin or of Balsam of Peru 2 oz.
*b* Tincture of Benzoin 6 oz.
Fine liquid Turpentine 4 oz.

This kind of plaster is applied on white or black silk, stretched on a frame garnished with points. A solution of the isinglass is to be made in boiling water: to this is to be added the alcohol and tincture of benzoin, (*a*) mixed together hot and well filtered. Of this solution a thick coat is to be applied to the upper surface of the silk, by means of a brush or pencil. This coat being dried, five others are to be successively applied; afterwards two coats of the tincture of benzoin (*b*) and the turpentine. This last application increases its flexibility; and though some pharmacians prefer the tinct. bals. Peru, yet the latter scales off more readily, while it is more agreeable.

*Dover's Powders.*—The following formula for this ancient and celebrated powder is from the French Codex:

R. Sulphate of Potassa,
Nitrate of Potassa, each 4 grammes, or 61 76-100 troy grains.
Ipecacuanha in powder,
Opium purified,
Liquorice in powder, each 1 gramme, or 15 44-100 troy grains.

It is recommended, in the pharmacopœia of Swediaur, to melt the nitrate and sulphate of potash together in a crucible, and then unite them to the other powders. The dose is directed to be 12 grains.

*Cough Lozenges of Tronchin.*

R. Powdered Gum Arabic 8 oz.
Brown Hydrosulphuretted Oxide of Antimony,
Anise, each 4 scruples.
Extract of Liquorice 2 oz.
Gummy extract of Opium 12 grs.
White Sugar 2 lbs.

Mix and make into lozenges weighing 6 grains each. Of these, one may be taken occasionally in diseases of the throat and chest.

*Lozenges of Oxalic Acid, for Thirst.*

R. Pure powdered Oxalic Acid 2 dr.
Sugar 1 lb.
Volatile Oil of Lemons 20 or 30 drops.
Mucilage of Gum Tragacanth q. s.

These lozenges may be colored red by means of a little carmine, blue by Prussia blue, or yellow by tumeric, if desirable. They are very pleasant in fever. And if it be desirable merely to make an *oleo saccharum*, the mucilage need not be added, and the compounds can be preserved in the state of powder, and used to prepare *lemonade.—Phil. Jour. of Pharmacy.*

20. *Effect of Light on Plants.*—By M. Leuchs. It is well known that solar light, by enabling plants to decompose and assimilate carbonic acid, gives them the power of forming volatile and aromatic principles, and of acquiring a green color. Its presence is so necessary to flowering and fructification, that ripe seeds have never been obtained in darkness; on the contrary, if an etiolated plant be exposed for three, four, or five hours to the sun, it immediately becomes of an equally intense green color with those which have continually grown in light. Plants raised in the open air, when put into darkness, become pale, and fade in two or three days; those which, after being raised in darkness, have been exposed for a time to sunlight, cannot again support the privation of light, but die; and water charged with camphor, or essential oil, which has great power of invigorating plants, cannot prevent their destruction. The perfect absence of light is therefore very injurious to plants, and M. Leuchs concludes, that, without the light of the moon and stars, nights would destroy vegetables.

The light of a lamp can, although imperfectly, replace that of the sun; the plant becomes green, and tends to the light. When seeds were germinated in three vessels, the first uncovered, the second covered with single, and the third with double paper, those of the first vessel exhibited less external development, but when dried, they gave more solid matter; those in the second were more developed, but were more aqueous and loose; the difference was still greater in the third vessel.

The texture of various plants appears to be more or less aqueous (if the word may be used) when deprived of light, according to the nature of the plants. When plants were placed in a damp cellar or cave, enlightened by a flame, those nearest the flame contained most solid matter; the results were so regular, as to present something like a law, relative to the action of various quantities of light on vegetables.

Light reflected by mirrors appeared to have a very beneficial influence upon plants, and M. Leuchs thinks that many hill sides are rendered fertile by the similar reverberation of light from the neighboring rocks.—*Archiv von Castner.*

21. *Remarkable Case of Re-union of a divided Part.*—A case of this description, which occurred at a Quebec hospital, is worth recording.—A man, in chopping wood, cut off the first phalanx of the middle finger. For two hours after the accident, he remained occupied at home. Although the divided part of his finger then appeared to be deprived of vitality, it was determined to follow the plan of Mr. Balfour, and to attempt to re-unite the parts. The tip of the finger was fixed to the stump by adhesive plaster, and in three days union had taken place in two or three parts, and the extremity of the finger which had been divided had as much sensation as any other part of the body. The dressing was continued, and in three more days the re-union was complete.—*Bost. Med. and Surg. Jour.*

22. *Chorea.*—M. Dupuytren is in the habit of treating chorea with the cold bath and cold affusions. Whilst in the bath, violent spasms and very disagreeable sensations are excited; these soon pass away. Active exercise is recommended for half an hour, after each bath.—The daily repetition of this process, for a few weeks, usually cures the most obstinate cases.—*Ib.*

23. *On Mr. Wardrop's method of treating Nævus.*—Sir,—I transmit you two cases, one of nævus, the other of aneurism by anastomosis, successfully treated by the Kali Purum, as recommended by Mr. Wardrop in the former disease.

These observations will not only confirm the observations of Mr. Wardrop, but they will lead me to make some remarks upon the difference of the mode of action of this caustic, and that of nitrate of silver, which has erroneously received the same denomination. The first case, or that of nævus, will only require to be briefly and simply detailed.

The case of aneurism by anastomosis, was the subject of various chirurgical transactions by a surgeon physician, who, confounding the effects of the nitrate of silver with those of caustic, had long endeavored to cure this affection, by that remedy in vain.

The nævus was situated under the left side of the inferior maxilla, rather deeply seated beneath the skin, of the size of a walnut, but daily increasing in its dimensions: the patient was a child two years old. Having protected all but the central part by means of adhesive plaster, I applied the caustic potass to this part, in the manner directed by Mr. Wardrop. Ulceration was produced, and spread to the destruction of the nævus: a common poultice being applied, the process of destruction, which extended but to the boundaries of the nævus, was followed by that of cicatrization.

The case of aneurism by anastemosis occurred in a Mrs. Taylor, aged 34, occupying the middle part of the left ala of the nose, and appearing to penetrate through the textures of which it is formed. It had been subjected to scarifications both within the nostrils and externally, and to applications of the nitrate of silver during a period of eighteen months, without the slightest advantage.

I applied a small portion of caustic potass over the part externally, confining its operations by means of an adhesive plaster pierced in its centre for the purpose, after the manner of making an issue. This process was required to be repeated five times, at intervals of about five days. The aneurism being now destroyed, the part healed spontaneously, leaving a cicatrix which is scarcely visible, and no orifice through the ali nasi, as it was feared any cure must do.

I think it important once more distinctly to state, that the nitrate of silver is not a caustic in any sense of the word. It subdues inflammation, and induces resolution and the healing process. It preserves and does not destroy the part to which it is applied. The pure potass, on the contrary, is a caustic; it destroys; it induces the ulcerative process. Touch a part with the nitrate of silver—the eschar remains for a time, and then falls off, leaving the subjacent part healed. Do the same thing with the kali purum—it induces a slough, which, being separated, leaves an ulcerated surface. If an ulcerated surface, secreting pus, be touched by the nitrate of silver, the discharge is immediately converted into lymph. It is the property of the caustic potass, on the contrary, to induce not only ulceration, but suppuration.

In short, the peculiar properties of the nitrate of silver have long been kept unknown to us by its designation of the lunar caustic, affording the most striking instance of the influence of a term or of classification upon the human mind. The nitrate of silver and the caustic potass (as, indeed, all *caustics*) are as the poles to each other—the first preserves, the second destroys; the first induces cicatrization, the second, ulceration. JOHN HIGGINBOTTOM.—*Lond. Med. Gaz.*

24. *Evacuation of the Humours of the Eye by a Wound, with Restoration of Sight.*—This very interesting and remarkable case is related by Baron Larrey in his Clinique Chirurgicale, a work just published, and from which we shall extract some other instructive cases. A sergeant of the fifth regiment of the Royal Guard, fell on the morning of the 20th of March. 1821, against a pile of muskets, and struck his left eye against one of the locks ; the globe immediately burst, and was almost entirely emptied ; the man mechanically carried his hand toward the eye, and found in it some glairy matter, in the middle of which was a whitish globular body, which acording to all appearances, was the lens. He washed his eye with cold water, covered it with a bandage, and was carried to the hospital de la Garde Royale, a few hours after the accident. On examination M. L. found the eyelids ecchymosed, the conjunctiva red and swelled, and the globe collapsed ; there was a wound at the inferior half of the cornea, through which tbe iris was prolapsed, and from which there was a constant discharge of an albuminous fluid mixed with blood ; the bottom of the eye was of a dark red colour. The patient complained of violent pain in the orbit and the whole left side of the head. The eye was washed with cold water ; the iris, the internal margin of which was torn from the ciliary ligament, was reduced with a golden stilat, the edges of the wound in the cornea were brought into contact, the eye kept closed, scarification made in the eyelids, and blood taken from the temporal artery. The patient was placed in a dark room, took cooling draughts, and had ice applied over the head, and sinapisms to the feet. Under this treatment the pain in the orbit and head-ache subsided, and he had about an hour's rest. Towards evening he became feverish and was again freely bled. The ice and cooling potions were continued. On the next morning, symptoms of cerebral inflammation having manifested thomselves, he was cupped between the shoulders ; the other remedies were continued, and the bandage not removed before the ninth day, when M. L. found, to his great surprise, the globe almost of its natural size. The aqueous humour still escaped through the wound, on the edges of which there was some irregular membranous fragments, probably of the membrane of the aqueous humour, which were removed by the scissors. The patient said he could distinguish the light. The eye was again closed and covered with a compress, dipped in camphorated wine, and an aromatic poultice applied to the left temple and supra-orbital region. The other remedies, as well as the application of ice were continued, and the patient repeatedly cupped. On the sixteenth day, the bandage over the eye was again removed ; the pupil was irregular, the iris motionless at the inner and lower portion ; but its external half contracted perfectly well ; its interior margin presented a semilunar fissure of a line and a half in breadth. The patient said he could distinguish objects very well, though they appeared to him as if divided longitudinally. The eye was now again covered with a simple bandage, and daily washed with a decoction of poppy-heads, containing a small quantity of camphorated wine. Under this treatment the inflammatory symptoms gradually disappeared. On the twentieth day after the accident, the wound in the cornea began to cicatrize, and after a few days more, was perfectly healed. From this period the globe recovered its former size and form ; the pupil was drawn towards the temple, but perfectly sensible at its outer portion. Sight was perfectly restored, except that the patient was obliged to use concave glasses, and that when he looked at objects before him, they appeared to him to be divided. On the 15th of May he left the hospital to resume his duties as a sergeant, and on the 29th of the same month he was presented to the Societe Philomatique.

25. *Case of Tetanus, caused by a Foreign Body imbedded in the Ulnar Nerve.*—A young man was struck with a whip over the arm, so as to produce a slight wound at the anterior surface of the forearm, just over the ulnar nerve. This soon healed, and left a small elevated cicatrix. A short time afterwards, however, the individual was brought to the Hotel Dieu affected with tetanus, the cause of which could uot be ascertained. The case ended fatally, although the most extensive antiphlogistic treatment was employed. The post mortem examination gave no clue respecting the origin and termination of the disease; the brain, with its membranes, as well as the spinal chord, were perfectly healthy, &c.; it occurred, however, to M. Dupuytren, to examine the cicatrix on the forearm, in which he was surprised to find a knot of the whip imbedded in the substance of the ulnar nerve, the irritation of which had undoubtedly been the cause of the tetanic affection.

26. *M. Dupuytren's Treatment of Hernia Humoralis.*—Many patients affected with inflammatory swelling of the testicles after gonorrhœa, have lately been admitted at the Hotel Dieu. The history of the cases individually are of little interest, on which account we abstain from giving them; and only advert to the subject to notice the remarks made by M. Dupuytren as to his general treatment of such affections. He directs from twenty to forty leeches to be applied on the scrotum, as nearly as possible on the part corresponding to the testicle; and this application is repeated two or three times, if necessary. Fomentations, poultices, low diet, and dilulent drinks, with confinement to bed and the maintenance of the testicles in the support of a suspensory, are rigorously adopted. M. Dupuytren never, on any occasion, practises the method recommended by some, of introducing a bougie into the urethra, by way of irritant, and suffering it to remain there;—a sound or bougie so used, seldom restores the discharge, while it often augments the irritation of the testicles, sometimes even causing formidable mischief. Thus he has seen cases in which individuals have died of other diseases during the time they were under treatment for swelled testicles, by means of bougies left in the urethra: in them the vasa deferentia and vesiculæ seminales were filled with pus.

The result of M. Dupuytren's practice has been very successful, seldom requiring more than a few days for its completion; whereas the former method often lasted six weeks or two months.—*Lon. Med. Gazette, March,* 1830, *from the Jour. Hebdom.*

27. *Excision of the Upper Jaw.* By John Lizars, Esq.—The third operation was performed on the 10th of January last, on a healthy woman, aged fifty five. Eight months before this period she began to feel pain in her right temple and eye, the latter of which soon protruded in a slight degree, and immediately afterwards the cheek; a fetid discharge began to flow from the nostril of the affected side, and a tumefaction of the hard palate to appear. When she arrived in Edinburgh, there was, on the right side, a distinct bulging downwards of the hard palate, an evident protrusion of the superior maxillary bone forwards to the face, and upwards to the eye, which organ had a weeping, inflamed, and swollen appearance; there was also a small, round, firm tumour in the nostril, from which issued a sanious discharge.

Having prescribed a cathartic the day before, he operated as follows. Having placed his patient on a table, he first secured on the right side the trunk common to the temple and intermaxillary arteries, immediately below the posterior belly of the digastric muscle, slit up the right nostril close to the mesial septum, likewise the upper lip at the labial fossa, and the cheek from the angle of the mouth to the masseter muscle. He next divested the bone of its soft coverings, at the points where the saw was to be applied, by dividing the mucous membrane which invests the floor of the naris, also the gum from the naris to the palate, the palate backwards to the velum, and across to the dens sapientiæ, carefully preserving the adhesion of the velum to the palatine plate of the os palati; after which he dissected up the flap of the cheek from the superior maxillary bone towards its nasal process, and to where it forms the floor of the orbit, also outwards towards the cheek bone, and around to its bulbous process.

The superior maxillary bone being thus divested of its soft coverings, or cleared of these where it was intended to be removed, he applied the saw at the following different places, namely, the front of the bone between the naris and mouth; the palatine plate backwards from this, parallel with the longitudinal palatine suture: to near where the transverse palatine suture exists: across the same palatine plate towards the bulbous process; upwards between the bulbous process and the pterygoid processes of the sphenoid bone, across where it joins the cheek-bone; and, lastly, at its nasal process, parallel with the inferior margins of the lachrymal and nasal bones. He then, with strong scissors, cut the connexions of the orbitary plate with the os planum of the ethmoid bone, and orbitary process of the palate bone, deep into the orbit, to the spheno maxillary fissure; and was, lastly, able, by notching with the bone forceps at every point where the saw had been applied, to remove the entire bone, which had its cavity filled with a firm sarcomatous tumour. There were, besides this, two or three soft gelatinous polypi hanging from the mucous membrane of the ethmoid bone, which required to be removed. A little lint was then inserted in the vacuity to afford support to the flaps of the cheek, which were approximated by needles and ligatures, three needles being inserted in the integuments of the nose, two in the upper lip, and four in the cheek. The patient was now carried to bed, and had an opiate administered. The wounds of the integuments healed by the adhesive inflammation. The patient was able to walk about the room by the eighth day, and the 5th of March she left Edinburgh for her home.

This operation appears hazardous, but Mr. Lizars says that it is not so in reality—that when once the hæmorrhage is commanded by securing the internal maxillary artery, it is comparatively easily effected, and not nearly so formidable as the former operation of trephining and cauterizing the antrum. The disfiguration of the countenance, he says, also is rendered very trifling by the preservation of the cheek bone.

28. *Tapping in Hydrocephalus.*—Dr. Conquest is said to have performed the operation of tapping for the cure of hydrocephalus, twice with success; and it is stated in the London Medical Gazette, for April last, that he exhibited one of the patients to his class, and a number of gentlemen, last spring. "This child, a girl of about two years of age, had several signs of hydrocephalus from a date soon after its birth, and for many months past the head had gradually increased, until it acquired an enormous size. The forehead was singularly broad, and the anterior fontanelle unnaturally large. The pupils were permanently dilated; the child slept almost incessantly, and frequently had two or three frightful convulsions during the day and night. Dr. Conquest operated sometime since, before a large number of the pupils of the hospital, by pushing a very beautifully constructed trocar into the right lateral ventricle. He introduced it obliquely, close to the edge of the right frontal bone, about midway between the crista galli process of the ethmoid bone and the anterior fontanelle, so as to avoid the longitudinal sinus on the one hand, and the corpus striatum on the other. The instrument entered about two inches below the scalp. An ounce and a half of bloody serum, mixed with portions of cerebrum, escaped. The pulse became feeble, and temporary collapse followed. The fluid was allowed to escape stillicidium, and within eight and forty hours about two pints and a half flowed out of the opening. Almost immediately after the operation, the pupils became sensible to the stimulus of light; the drowsiness was succeeded by disinclination to sleep, and the pulse, which had always before been remarkably slow, became about eighty-five. Two days after the operation, the brain evidenced signs of inflammation, with high constitutional disturbance; and great alarm was excited by a rather formidable attack of convulsions. Leeches to the temples, and the constant application of cold to the head, subdued the local inflammation, and within four and twenty hours all became tranquil. The head was well strapped, and from the cessation of cerebral excitement no unfavorable circumstance occurred.

"When this interesting child was exhibited to the class on Saturday evening, every one was struck with the improvment of its appearance, and by the intelligence and cheerfulness of its countenance.

Dr. C. stated that he considered it perfectly well, and as exhibiting a most gratifying and triumphant proof that this seemingly formidable proceeding might be safely and successfully adopted under similar circumstances.

"The other case, to which the doctor has often adverted during the winter, he operated on last autumn, assisted by Dr. Hodgkin, the talented pathologist of Guy's Hospital. Nine ounces of serum were withdrawn from the posterior fontanelle. The head became lessened six inches in its circumference, and no increase of its size has yet recurred."

29. *Case of Recovery from a Wound in the Stomach, inflicted by a Pistol Ball.* By P. Breton Esq.—A trooper, in a fit of despondency, formed the resolution, on the 30th of April, 1819, to commit suicide. In the evening, about dusk, he took his holster pistol, and three ball cartridges, and proceeded from the fort of Sembhelpur to the bed of the Mahanadi River, where he intended to effect his purpose. His woman, seeing him in a disturbed state of mind, followed him at a short distance. Observing him load his pistol, aud directing it towards himself, she ran and seized his arm, and endeavored to force the pistol from his hand. In the struggle, the pistol was fired, and the ball passed through the body of the woman, and killed her on the spot. The trooper again loaded, and shot himself in the pit of the stomach, the ball lodging in the left side of the lumbar vertebræ.

Having failed in his attempt to destroy himself, and the third ball cartridge with which he had provided himself being lost when he fell on the ground by the shock from the ball, he returned, without any assistance, to the fort, and communicated every thing that had happened.

"On examiniug his wound, I found that the ball had entered the epigastric region, and was perceptible to the feel in the left side of the loins. I immediately extracted it, and closed the incision with adhesive plaster. Conceiving that the stomach was penetrated by the ball, I considered the case a hopeless one, and reported the wound to be mortal. The following morning the man had recovered his senses so as to give a distinct and rational account of all the circumstances which led to the commission of the rash act; and the wound manifested such appearances, as left no doubt in my mind of the stomach being perforated.

"My attention was therefore directed to uniting the anterior wound with all possible speed, and to prevent the introduction into the stomach of any more food than was absolutely necessary to support life. To my astonishment, the wound quickly united, and the serous discharge from the stomach gradually ceased. When union had taken place in the anterior wound, I was informed by the native doctor, that when food was taken into the stomach, particles now and then escaped from the wound into the loins, where the ball lodged. To convince myself of the truth of this assertion, I gave the trooper a draught of water to drink; and almost immediately afterwards, I distinctly saw a small quantity of it trickle through the wound. I repeated this two or three times, and was satisfied that there was a communication between the stomach and the wound in the loins. Superficial dressings were continued, and rigid abstinence enjoined. In the course of about a month and a half, the wounds healed, and the man recovered."

It is remarkable, that in this case of perforation of the stomach by a pistol ball, very little pain and uneasiness were expressed by the patient, and symptoms of irritation were not at any period manifested. Neither was thirst complained of, nor were the natural functions of the body in any considerable degree disturbed.—*Transactions of the Med. and Phys. Society of Calcutta, Vol. I.*

30. *On the Influence of Electricity on Animal Putrefaction.* By Charles Matteucci.—Persuaded from some experiments of M. Bellingeri of Turin, and others not yet published which I have myself made, that animal substances, when they are put in contact with metals, establish themselves in an electric state, I determined to place some pieces of muscle upon plates of zinc, others on plates of copper, and I left others by themselves. In the course of a day I perceived that putrefaction had already begun in the pieces of muscles which were left to themselves, while no alteration showed itself in those which were in contact with metals. I afterwards perceived that the products of the change which had afterwards taken place on these last were different, but were always related to the electric state which they had assumed, that is, with their affinity. I observed, for example, ammonical products, and those of carbonated hydrogen in the muscles which were in contact with the zinc; and much acid and acetate of copper in those which were in contact with the copper. These results prove sufficiently that muscle, put in contact with zinc, having become electro-negative, and being no longer able to unite with the oxygen, have been slow in decomposing; but have at last yielded to the affinity, though weak, of the hydrogen and the azote, while on the contrary, the muscular fibre, placed on copper, were combined entirely into acid products. We may then, in this manner, retard putrefaction, that is, by eluding the action of one of the two elements of the atmosphere. I have thus obtained similar and perhaps more marked results by determining an electric state in the animal fibre, not by electro-motive action, but by placing them as conductors at the poles of a pile.

By setting out with these considerations, it appears to us that we may, with more certainty, explain the antiseptic property of some bodies, an explanation, however, which is not the same for all. There are some, for example, which act by taking away water, others by forming true imputrescible combinations; others, in my opinion, by determining a particular electric state. Of this kind is the property of vegetable charcoal. It is a settled fact in practical surgery, (as has been shown by Dr. Palman in a pamphlet lately published in

Paris) that if we put vegetable charcoal on purulent sores and on putrid sores, it is not long in depriving them of their bad smell, and preventing the ulterior development of fetid matter.

Effects like these cannot depend solely on the action of prorosity, for they would cease by continued contact ; and we may explain them better by regarding the action of charcoal as electrical, in consequence of which, by establishing in purulent sores, and in putrid flesh, electrical states, they lose those affinities in virtue of which they separate the purulent matters, or destroy them by a rapid putrefaction.—*Annales de Chinie.*

31. *Purity of Balsam Copaiba.*—The best test of this, according to M. Gerber, is the caustic ammonia, which furnishes at once a clear solution, whilst the solution with potash does not become clear until after some time. The addition of a very small quantity of fatty oil renders the ammonical solution immediately cloudy and thicker.
*Ibid.*

36. *Transfusion.*—Dr. Dieffenbach, of Berlin, has performed numerous experiments upon animals, from which he obtanss the following conclusions :

1. That an animal, bled to apparent death, may be brought back to life by the blood of another animal of the same species, and afterwards continue to do well.
2. Blood derived from an animal of different species may produce signs of life, but these are not permanent.
3. If transfusions be performed with the blood of an animal very dissimilar to the one operated on, however small the quantity, the latter will be destroyed. This result occurred when human blood, or that of a calf, was injected into a cat ; more slowly when that of a dog or a rabbit was transferred to the veins of the same animal.
4. Mammiferous animals are less sensible to the noxious influence of the blood of birds or coldblooded animals, if they have previously been bled.
5. Birds are always killed by the blood of the mammifera or fishes, and exhibit, in these cases, the symptoms produced by narcotic poisons.
6. Whenever, after the injection of the noxious blood, an animal has copious evacuations, by urine, stool, or vomiting, the ill effects of the operation are lessened by this circumstance.
7. Blood, when exposed to the air, does not lose its reviving properties, until decomposition commences. Once decomposed, it produces the same effects as any other putrefying animal substance.
8. Age, sex, and other circumstances or states of the constitution, exercise little or no influence on the effect of transfusion.
9. Diseases are not always communicated by transfusion.
10. Venous blood answers best for this operation.

11. Transfusion is always attended with danger, although the animals are of the same species ; its employment as a remedial measure, therefore, is suited only to desperate cases, where all other means are inadequate to preserve life ; and none except human blood ought ever to be used in these cases.—*Bost. Med. and Surg. Jour.*

32. *New Pessary.*—A lady practising the profession of midwife near Paris, has lately invented a pessary which, on some accounts, has been found Preferable to any heretofore employed. It consists of a steel spring of proper shape, enclosed in a cushion of horsehair, which again is entirely enveloped in a case of gum elastic. The instrument is said to have sufficient firmness to support the uterus, to yield readily to the pressure exerted during the fecal evacuations, and to oppose no obstacle to the passage of the urine. It has received the approbation of the Royal Academy.—*Boston Medical and Surgical Journal.*

33. *On the Use of the Acetate of Lead in Ulcerated Phthisis.*—Dr. Lenz considers the acetate of lead as a true panacea in chronic pneumonia which has gone on to ulceration. Dr. Schneider, of Ettenheim, is also said to have employed the remedy in this disease, with success. The medicine is given in powder, combined with opium ; the dose gradually increased. A patient who was cured by Dr. Lenz, took two drachms in thirty-two days ; and Dr. Schneider has often given fourteen grains in one day, without producing the least ill effects.—*Heidelb. Klinis. Annalen.*

34. *Fracture of the Spine—Recovery.*—A case of recovery after fracture of the spine, with paralysis, recently occurred at St. Thoma's Hospital, and is reported in the London Journals. The subject of it was a young man 15 years of age. The fracture was produced by the falling on him of a cask, and was discovered in the body of the 3d dorsal vertebra. Although the mind was perfectly clear. and the use of the upper extremities unimpaired, respiration was performed by the diaphragm alone, and there was total loss of voluntary motion in the lower extremities : prinapism was constant, and the patient had no power over the bladder.

He was placed upon a fracture-bed, with a trap door so fixed that the seat of the injury might be examined without moving him ; leeches were applied freely, the catheter, enemata, &c., used as occasionally indicated The amendment was gradual, and it was not until he had remained on the bed twelve weeks, that he was allowed to rise. At the expiration of five months from the accident, his health was restored, and he walked perfectly well, save only a degree of stiffness in the hip, knee, and ankle joint, of the right leg.

35. *Physiology. Seat of Taste.*—From an account of experiments made to determine this point by Messrs. Guyot and Admyrault, of

Paris, we select the following, which will be found to present some very curious results:—

1. If the extremity of the tongue be enclosed in a sac of soft parchment, in such a manner as completely to cover it, it will be possible to introduce between the lips, and even to crush between them, a small quantity of a soft sapid substance, like preserves, without producing any other sensation than that of consistence and temperature. This experiment has been varied by employing weak hydrochloric acid, and sweetened water, without its being possible, not only to distinguish them, but even to recognise any taste in either.

2. If the cheek be withdrawn from the alveolar arch, and then be covered internally with sweet or acid jelly, no sensation of taste is experienced, unless the saliva from the part is allowed to come in contact with the tongue. This experiment may be varied by simply closing the teeth, and placing between them and the cheeks a soluble body, like sugar or aloes. The taste is not perceived, even when the substance thus enclosed has been dissolved; but it becomes at once obvious, when the fluid of the part is allowed to flow within the teeth.

3. The tongue being covered as in the first case, but to a greater extent, by adding a prolongation reaching to the epiglottis, if some substance of a decided taste is swallowed, and, in the movement of deglutition, is carefully brought in contact with all the points of the palatine arch and the velum, the taste will be perceived on the posterior part only.

4. If the palatine arch be covered with a slip of parchment, a sapid body placed on the tongue and swallowed, produces its usual taste.

5. A fragment of aloes fixed on the end of a stilet, and placed in successive contrast with all the points of the arch and the velum, produces the following effects:—In the whole extent of the arch, at its edges as well as the centre, there is no sensation but that of touch.—The same is the case generally with the velum, and with the greatest part of the uvula. It is observed, however, that, in the anterior superior part of this organ, about a line below the point of its insertion into the arch of the palate, there exists a small surface, not extending to the base, from which it is three or four lines distant, but prolonged and losing itself laterally; this part is endowed with the sense of taste in a marked degree. By carrying the instrument farther into the mouth, it appears that the posterior part of the velum and the mucous membrane of the pharynx, have no part in producing the sense of taste—If, then, we except the part just indicated at the superior part of the velum, the tongue is to be regarded as the sole seat of taste.

8. A stilet, armed like the one last mentioned, and applied in succession to different points of the tongue, will prove that the whole face of this organ does not possess the property of perceiving tastes. This property is found only as we approach the circumference, in a region of one or two lines in breadth along the sides, and three or four at the point, somewhat broader on the superior than the inferior face. It is also found to exist in the space situated beyond a curved line passing through the blind hole, and the concavity of which is supposed to be turned forward.

The sense of taste is possessed by the border of the tongue more fully than by any other portion, and appears to exist uniformly in this part, as far as a few lines from the anterior extremity. From this point the sensation becomes more acute, and reaches its maximum at the point, where it exists in the greatest intensity. With the exception already noticed, the inferior surface of the tongue is incapable of taste.—*Bost. Med. and Surg. Jour.*

36. *Case Illustrating the Intoxicating Effect of Sulphate of Quinine.* By Chandler Robbins, M. D.—The following history displays not only the influence of quinine over the cerebral functions but shows us in what quantity some constitutions (and perhaps all) will bear a remedy which is usually prescribed in doses of two or three grains.

On the 5th of January, I was requested to visit Mr. O., a young gentleman about 30 years of age, with a constitution naturally good, but health somewhat impaired by frequent attacks of intermittent fever. He first contracted this disease on the canal route to Saratoga; and for many months had been in the habit of arresting the paroxysm at its onset, by a liberal dose of sulphate of quinine, which almost uniformly effected his purpose, if taken immediately on the approach of such sensations as he had learnt to consider premonitions of an attack. The day previous to my visit, such sensations having been rather more urgent than usual, he took *half a drachm* of the sulphate at a dose. This quantity produced no stricture across the chest, and no pain in the stomach; but in about three quarters of an hour he began to feel as if intoxicated. This feeling, which was precisely similar, he states, to that produced by an over dose of alcohol, increased so rapidly, that in about fifteen minutes his ideas became confused, and he was unable to walk without staggering. He succeeded, however, in getting his bed and some sleep, and the next morning complained only of loss of appetite, occasional dull pain and dizziness of the head, and a general languor and debility, and incapacity for business;—his symptoms, in fact, were such as are usually the sequelæ of inebriation, but more permanent, yielding but tardily to the remedies prescribed. This gentleman had no return of intermittent for a long time after, nor do I learn that it has ever troubled him since. He is now an excellent editor of an excellent newspaper in the state of New-York, and will probably recognise his case in the above details. *Franklin Place, Boston.* —*Bost. Med. and Surg. Jour.*

37. *Distinctive marks of Pregnancy.*—Since pregnancy may exist with but questionable evidence, and, on the other hand, all its imputed symptoms may be present under circumstances foreign to gestation, it becomes an interesting and practically important inquiry, are there any means of arriving at the truth, in every such case, at a period antecedent to parturition.

No one, it appears to us, has answered this question so well as Dr. Gooch, who, in his late account of some of the principal diseases peculiar to women, has given to it about fifty pages. His opinion appears to be, that, when we are in doubt, nothing can solve it so readily as a careful external or internal examination,—each separately, and both combined.—The directions given by Dr. Gooch for performing these examinations, we shall offer in his own words.

"*External.*—In examining externally through the walls of the abdomen, the bladder should be empty, the patient in bed, *in* her night dress, *on* her back, *in* a posture between sitting and lying, with the knees slightly drawn up. These are the most favorable circumstances for the external examination; but we are often obliged to examine without these advantages.

The first thing to notice is the situation, consistence, and figure of the tumor which is distending the abdomen. In pregnancy, the uterus does not rise out of the pelvis before the third month,—by the sixth month it is up to the umbilicus,—by the seventh it is a little above the umbilicus,—by the eighth month it is half way between the umbilicus and scrobiculus cordis,—and in the ninth month it has reached the scrobiculus cordis, its highest elevation; thus, if we are examining a patient about the sixth month of pregnancy, we shall feel a circumscribed tumor occupying the front of the abdomen, from the brim of the pelvis to the umbilicus, of an oval form and firm consistency, much firmer than the abdomen above and on its sides, where it is occupied by the intestines. All this can be made out clearly, if the walls of the abdomen are thin and relaxed; if they are fat, this is difficult, and often impossible; but even then we can notice whether the enlargement is firm or soft: the former will be the case, if the patient is pregnant.

The next thing to notice is the umbilicus. In the unimpregnated state, it is sunk below the surface, forming a shallow pit; but in pregnancy, when the uterus has arisen to or above the umbilicus, this part projects above the surface of the abdomen: this, however, depends on the period of pregnancy at which we are examining; it will scarcely be found before the sixth month, and the further the pregnancy is advanced, the more distinct will it be. The firmness of the abdomen, and the projection of the umbilicus depend on one and the same cause; that is, the firmness of the tumor which is distending the abdomen; but any other tumor equally firm may occasion both of these symptoms: their presence alone proves little, but if the state we are investigating is advanced as far as the seventh or eighth month, their absence proves a great deal; for, if the umbilicus is depressed, and the abdomen, though enlarged, is soft and yielding, these alone prove that the patient is pregnant. Let not the practitioner, however, give an opinion till he has collected all the proofs."

Before proceeding to internal examination, one other point is of great moment. The hand should be placed on the abdomen, over the tumor, and there allowed to remain several minutes. If no motion is felt, it is not indeed conclusive evidence that there is no fœtus in utero,—the fœtus may be quiet just then, or it may be dead,—but if a motion *is* distinctly felt by the hand in this situation, this is perhaps the best of all proofs of the pregnancy of the patient,—much more conclusive, certainly, than any obscure sensation of the patient herself, who may mistake other movements, or even nervous impressions, for fœtal motion. Dr. G. then goes on to speak of the examination which we have called

*Internal.*—"Having examined the uterus through the walls of the abdomen, we proceed next to examine it through the vagina; for this the patient should be turned on her side;—and here again there are three things to observe,—the state of its neck, the state of its body, and the movement, or rather the mobility of the fœtus. 1st, in the unimpregnated state, the neck of the uterus projects into the vagina about two-thirds of an inch, like a thick, firm, fleshy nipple. At the termination of pregnancy, a few days before labour, this neck is completely obliterated, the portion of uterus which lies over the top of the vagina, no longer projecting into its cavity, but forming a flat roof. This obliteration begins about the fifth month, the neck becoming gradually softer, broader, and shorter; by the seventh month it is much altered, and not at all like the neck in the unimpregnated state, being very soft, broad, and short. It is now calculated to have lost three-fourths of its length; but it is not quite obliterated till the last week of pregnancy; so that if a false alarm about labour, two or three weeks before delivery, gives the practitioner an opportunity of examining the uterus, he will find a soft, short nipple still remaining."

After satisfying himself of the state of the neck of the uterus, the medical attendant should next ascertain if the body of that organ be enlarged, by placing the finger up between the os uteri and pubis. In its unimpregnated state, the uterus will be felt soft and flaccid; if distended, a hard tumor will be distinctly perceived in this situation.—We may then proceed to the

*Combined Examination.*—"There is," says Dr. G., "a combination of the external and internal examinations, which, in thin persons, gives a very accurate knowledge of the nature of the tumor. For this purpose, the finger of the right hand is to be applied against the tumor which is felt in the vagina, and the left hand is to be applied on the outside of the abdomen, to the upper part of the circumscribed swelling. Now by alternately dressing the tumor up, by means of the finger in the vagina, and down, by means of the hand on the abdomen, the practitioner becomes certain that the tumor which is felt through the walls of the abdomen, is the same as that felt through the vagina; the most satisfactory proof that it is an enlarged uterus. This method is applicable as early as the fourth or fifth month."

Before giving a decided opinion, one other expedient should be adopted. It is well known that the fœtus is not closely embraced, but always floats in the liquor amnii. Let the patient then be placed in an erect posture, and then applying the finger (in front and a little below the os uteri) to the uterine tumor, push it gently upward; if the tumor be a fœtus, it will follow the finger up and back again,—next give it a sudden push upward; if a fœtus, it will leave the finger and again come down upon it,—a circumstance which will not occur in any disease of that organ. This is, in fact, the most conclusive part of the whole examination, and when made between the fifth and seventh month, and taken in connexion with the result of the preceding trials, can seldom leave the attendant in doubt. We regret that our limits will not allow us to give other views of the diagnosis proposed by Dr. G. Those we have offered, if attentively considered, cannot but prove highly useful to every medical practitioner.—*Bost. Med. and Surg. Jour.*

37. *Artificial Eyes.*—Dr. Scudder, the ingenious oculist, whose artificial eyes are said to have pupils which contract and dilate, and to wink and move in the same direction with the natural organ, is now at the Tremont House in this city. We have seen specimens of his ingenuity, and, after attentive examination, would recommend them with great confidence to those who are so unfortunate as to require such an appendage to the natural phisiognomy. The artificial shell which is made to imitate the natural eye, is fixed by Dr. S. directly to the ball, and consequently partakes of its movements, and allows the lids to close over it. This is what is called making an "artificial eye to wink and move with the real one in any direction."

As to the contraction and dilation of the pupil, few could expect any great power or extent of motion in the internal part of a solid lens of glass. By an optical deception, however, the pupil, though perfectlystationary, does *appear* to alter its diameter with every motion of the enamel. As the latter changes its direction, the former *appears* to dilate, or contract, and this, though these changes be ever so trifling.—The manner in which this effect is produced, is as follows:—The pupil is represented by a black substance at the posterior part of a hemisphere of transparent glass, which occupies the place of the aqueous humor in the real organ. Around this substance the iris is represented,—not painted, but colored apparently in the glass itself. Thus viewing the black substance or pupil through a very convex lens, its size will vary according to the direction in which we look upon it. If we see it sideways, its size is greater than when the eye is cast upon it directly; and hence the apparent change in its size with every movement of the organ.

But further;—in some of the specimens we saw the edge of the pupil was indistinct, except in a strong light, and this indistinctness was produced by the deep shade of the inner edges of the iris. The effect of this is admirable. If we bring the eye to a strong light, the line of the circumference of the pupil is clearly distinguished: if now we remove it to a less powerful light, this line is not discerned, and being lost in the deep shade of the margin of the iris, the effect is an apparent increase of size, or, in other words, dilatation of the pupil itself. By this very brief and imperfect description, the general principles on which these machines are constructed may be learnt. Dr. S. has doubtless brought the imitation nearer to the original than any one who has preceded him; and this meed will doubtless be accorded him in Europe, whither he intends going in a few months.—*Bost. Med. and Surg. Jour.*

38. *Mode in which the Poison of Lead is conveyed into the system.*—It was stated by Dr. Thompson, at a late meeting of the Westminster Medical Society, that, from a number of experiments and observations, he had been satisfied that none of the salts of lead are poisonous but the *carbonate;* and that where mischief had resulted from the use of the acetate or subacetate, the cause was the conversion of these into the carbonate. When exposed to the action of the air or of pure water, lead, as every one knows, becomes tarnished. This tarnish is not an oxydation, but the formation of a thin crust of the carbonate which is formed on the lead; and if this crust be scraped off and mixed with acetic acid, brisk fermentation accompanies its solution.

In view of these facts, we should caution the public against the use of lead pipes to conduct soft water which is to be subsequently conveyed into the human stomach; and much care should be used in avoiding, for culinary purposes, rain or snow water which has passed over roofs or spouts lined with lead. Rain, snow, or spring water, are mentioned; for it has been ascertained by Dr. Christison, that the presence of neutral salts and other foreign ingredients held in solution by water, impairs its power of thus acting on the lead; and this is the reason why soda water, passing through pipes of this material, is so seldom poisonous. This caution is particularly necessary for persons who reside at a distance from the seaboard, and particularly in sandy regions, where most of the water used in cooking, &c., is comparatively destitute of these ingredients As for Boston, there can be little apprehension on this score; we can truly say as Dr. Johnson, when treating of the same subject, says of London, "if purity of water be a dangerous property, this metropolis is as secure as if the inhabitants drank nothing but nectar."—*Bost. Med. and Surg. Jour.*

39. *Division of the Submaxillary and other Nerves.*—Sir—I observe in your fasciculus for February last, the description of an operation performed by Dr. Warren, for excision of the submaxillary nerve, extracted from the Boston Medical and Surgical Journal. This mode of operating appears truly formidable, and is probably that which the late Dr. Haighton had in view, when he pronounced the division of this nerve impracticable.

A much simpler, safer, and easier mode of accomplishing the division of this nerve, where it enters the canal of the inferior maxillary bone, is, to make an incision, with a scalpel, from within the mouth to the extent of an inch; through the mucous membrane and cellular tissue connecting the pterygoideus internus muscle, to the ramus of the bone, parallel and close to the inner or mesial surface of the coronoid process immediately behind the dens sapientiæ; then to make a round-shaped gum lancet, and carry it backwards in a line continuous with the crown of the molar teeth, having the cutting edge at right angles to the bone, and divide the nerve on the bone. The pain experienced on the division of the nerve, at once indicates that the proper organ has been cut. As the internal maxillary artery ascends to the bulbous process of the superior maxillary bone, it cannot be wounded, excepting through ignorance or carelessness; but, even if it were, a piece of dry sponge might be easily inserted to stem the hæmorrhage. The gustatory branch of the nerve could scarcely be injured. The dental artery must be wounded, but this is so small as to be of no moment, and, if morbidly enlarged, dry sponge would compress it. I have now performed this operation on four patients for neuralgia of the mental nerve, with perfect success, having previously attended to the chylopoietic viscera, and then tried the various antispasmodics and subcarbonate of iron; also the different counter-irritants, even the moxa, and lastly, the division of the nerve as it emerges at the mental foramen. Or, according to your own showing,—"after the local symptoms from morbid associations or change of structure, had continued after the constitutional derangement from which they originally emanated, had been rectified—and the consequence had survived the cause." My first case was published in the Edinburgh Medical and Surgical Journal for October, 1821.

Thus there would appear to be a material difference between the division of the trunk of a nerve, where it is protected from the vicissitudes of atmospherical influence by muscular and other soft coverings, and the division of the same nerve, where it is exposed to the alternations of the weather, as far as relates to the permanent salutary result. It is well known, that in neuroma supervening to amputation, the excision of the tumour or tumours, proves a more permanent or radical cure than a secondary amputation, also in neuralgia following the same operation, excision of the nerves does the same, and evidently in consequence of excision preventing the interesting junction of the nerves, as well as the production of the numerous delicate filaments supplying the cicatrix of the stump. This has been satisfactorily described by Larrey, in his late valuable work 'Clinique Chirurgicale,' and also by Descot, in his interesting 'Dissertation sur les Affections Locales des Nerfs.' For the same reasons, the excision of a portion of a nerve must be a more effectual cure than simple division of the same.

If this view of the operative department of the pathology of nerves be found to be correct, it would follow, that the division of the infra-orbitary nerve, where it enters the osseous canal in the floor of the orbit, would prove more availing, than its division at the infra-orbitary foramen on the cheek.

This might be easily accomplished as follows:—let an incision about an inch long, of a curvilinear figure, to correspond with the circular shape of the orbit, be made at the outer canthus of the eye, the centre of which shall be opposite the outer commissure or angle of the eyelids, or rather the superior margin of the zygoma. This incision is to be deepened by cutting close to the osseous wall of the orbit, until the instrument reach the spheno-maxillary fissure, when it is to be laid aside, and a round shaped gun lancet inserted in the wound, with its cutting edge at right angles to the floor of the orbit, and the nerve divided as it runs in the osseous channel. In some, this is an open, while in others, it is a shut or entire canal; but, in all, the parietes are so delicate as to be easily cut across. A portion of the infra-orbitary nerve, at its emergence from the infra-orbitary foramen, could not be removed, in consequence of its division into so many minute filaments; neither could this be accomplished within the orbit.

The supra-orbitary or frontal nerve may be also divided within the orbit, nearly an inch from the superciliary ridge, by first ascertaining the superciliary foramen or notch, which is done by drawing a perpendicular line from the second bicuspis at right angles to the area of the crowns of the teeth; secondly, by making an incision about the fourth of an inch, parallel and close to the superciliary ridge at the foramen, through the integuments, orbicularis palpebrarum muscle, and ligament of the superior tarsus; thirdly, substituting for the straight bistoury or scalpel, a probe-pointed bistoury, which is to be insérted deep in the orbit close to the bone, and with which the nerve is to be divided by cutting upwards on the bone, in the direction from the inner to the outer canthus, carefully guarding against injuring the superior oblique muscle on its inner or mesial aspect. A portion of this nerve may be excised either within or without the orbit: within, as just directed, combined with searching for the nerve at the superciliary foramen, and after its division, seizing hold of it with the dissecting forceps, and removing the insulated or detached part. As it sends off minute filaments on its emergence from the orbit, the removal of a portion without the cavity, would not hold out a prospect of so permanent a cure. JOHN LIZARS.—*Medico-Chirurg. Review.*

40. *A Case of Labour in which the Placenta was delivered some time before the Fœtus.* By Dr. Caleb W. Cloud, of Lexington, Kentucky.—On the 3d of February, 1830, at 11 o'clock P. M. I was called to visit a woman, who was said to be in labour. I found her in considerable pain, with great uterine hæmorrhage; she said her labour was premature, and that she was in the eighth month of her pregnancy. On examination per vaginam, I found the os uteri considerably dilated; one knee of the fœtus, and a part of the placenta could also be distinguished. The pain subsiding, I gave her syrup of rhubarb and laudanum, and left her for the night.

At 8 o'clock A. M. of the ensuing day, I saw her again, when the pain was slight, though the hæmorrhage continued. At 12 o'clock M. the uterine pain was increased and the bleeding had become quite alarming. On examining again I found the membranes protruded, which were soon afterwards ruptured, when a large portion of the placenta together with the umbilical cord, presented. Pulsation was felt in the cord, but no portion of the child could be reached

In this situation, I gave her fifteen grains of ergot, and after an interval of twenty or thirty minutes, the same quantity was repeated.

In half an hour from the exhibition of the last dose, the placenta was expelled, and the hæmorrhage immediately ceased. Still the child could not be felt, per vaginam, and I had some apprehension at the time that it might have escaped into the cavity of the abdomen, by a rupture of the uterus. The woman, however, being a good deal fatigued, was permitted to rest for two hours or more; when, there being no return of pain, or descent of the fœtus, I gave her fifteen or twenty grains of ergot, and repeated the same quantity in twenty minutes. Some pain now came on; but, being slight and ineffectual. I introduced the hand and dislodged the child from the os pubis, on which it rested, inclining to the right side: after this I was enabled to reach it with the forceps, (the breech presenting,) and in this way it was delivered.

After delivery the woman complained of great debility, with sick stomach and vomiting—these, however, were soon relieved and she recovered speedily. The child was well formed and of the usual size. The mother is about 37 years of age; this was her eighth pregnancy, all the preceding labours being natural; and she knows not of any cause to have produced premature or preternatural labour in the present case.—*Transylvania Jour. of Med.*

41. *Observations on a Peculiar Kind of Traumatic Delirium.* By M. Helis.—The object M. Helis has in view, is to illustrate by cases the advantage of the treatment recommended by M. Dupuytren, which we have ourselves seen employed with the best effect.

Case 1.—*Delirium after the operation for Sarcocele.*

M D. R., twenty five years of age, of a nervous temperament, was operated upon for sarcocele of a very large size. in 1817. The day after the operation he was restless, and very much alarmed lest hæmorrhage should occur. The succeeding day his agitation had much increased; the slightest word or movement produced the greatest degree of excitement; the least sensation redoubled his apprehensions. Still his progress was satisfactory. He soon complained, however, of pains in his limbs and chest; his eyes glistened, he breathed quickly, eagerly demanded food, and was determined to rise from bed. His mind wandered; he repulsed those who were the most attentive to him, and called loudly for his family; his whole body was incessantly in motion. His cries, the appearance of his eyes, the fixed state of the pupil, his face covered with sweat, and his calm and regular pulse during all his agitation, convinced M. Dupuytren of the nature of the case. Particular attention, however, was paid to the state of the chest, as the patient complained of a fixed pain in that part.

When it was ascertained that he was laboring under no pulmonic disease, an enema with six drops of laudanum was immediately given; and the patient was ordered to be kept perfectly quiet, and undisturbed by friends. In an hour after the administration of the enema, M. D. R. ceased to ramble, and fell into a sound sleep, which lasted for several hours. The cure was complete in twenty-five days.

Case 2.—*Delirium after Luxation of the Femur.*

A mason fell from a scaffold, and luxated the left femur. He was carried immediately to the Hotel Dieu, and the next day the luxation was reduced with the utmost facility. The patient was dreadfully alarmed at the apparatus which was employed, and could not believe himself so speedily cured. On the following day he was extremely agitated. The eyes were unnaturally brilliant, and turgid with blood, his face red, and covered with sweat. He cried out incessantly, and endeavored to break the bandages by which his limb was secured. In the midst of this mental derangement, the pulse was full, regular, and natural in number ; temperature of the skin not increased.

The sister of the ward, who was well accustomed to these symptoms, ordered an enema with ten drops of laudanum in it to be immediately given ; and no other remedy was necessary to restore the patient to reason.

Case. 3.—*Delirium after the Fracture of a Rib.*

Langlois, a mason, was admitted into the Hotel Dieu with fracture of a rib. A tight bandage was placed round his body. From the facility with which such accidents are usually cured, but little attention was paid to this patient ; but on the third day he was attacked with delirium, which continued unabated. He threw himself into various positions : all his muscles were in a state of continued tension ; eyes very brilliant ; skin covered with sweat ; pulse natural. He fancied he saw images dancing before him in the air, and that various experiments were being tried upon his bed.

As this man was of a full habit of body, he was first bled, but without relief. An enema with ten drops of laudanum was then given, and a slight degree of calmness succeeded. The next day this dose was doubled, without advantage. His cries and constant restlessness were now so annoying to the other patients, that he was put in a ward by himself, and forty drops of laudanum were given in an enema, and the delirium speedily subsided.

Case 4.—This patient had attempted to commit suicide by cutting her throat. On the second day after the attempt he became delirious, and it was necessary to restrain him by the strait-waistcoat.

An anodyne was first given by the mouth, with but little effect ; but an enema with a few drops of laudanum quickly restored him to reason. On the fourth day the wound assumed a bad appearance ; the delirium returned, and was again successfully opposed by the same treatment.

Case 5.—This patient was operated upon for popliteal aneurism. He was of a plethoric and athletic constitution, and a free bleeding had been practised, to guard against accident after the operation. He appeared to be indifferent to every thing that was done, and scarcely conscious of what was passing around him. On the fifth day he was attacked with furious delirium, without fever. The symptoms were the same as those detailed in the previous cases. An Anodyne enema was given with the most complete success. The ligature, however, came away from the femoral artery prematurely, and the patient expired on the fortieth day, after many attempts had been made to arrest the hæmorrhage.

Case 6.—In this case the patient had attempted self destruction by cutting her throat. Delirium afterwards occurred, without fever or symptoms of inflammation. Anodynes, internally given, were effectual in relieving it.

M. Helis is not aware that any author has paid particular attention to this species of delirium. He has only found one example of it in books. Many surgeons have, indeed, spoken of the violence of some patients after operations, and of their tearing away their dressings ; but none have inquired into the cause of this kind of insanity, or have endeavored to relieve it by any other means than coercion. The danger of so violent kind of delirium after various accidents and operations, must be evident, and it must be a source of much gratification that we possess a remedy which is almost certain in its operation. M. Helis imagines that it may generally be possible to predict the occurrence of this mental disturbance, either from the nature or duration of the operation, the character of the patient, his moral energy or physical disposition. There are some symptoms from which delirium may be almost certainly anticipated. If, a few hours or one or two days after a fracture, an attempt to commit suicide, or any operation, the patient appears unusually gay, talks much, has a quick expression of the eyes, gives short answers, moves quickly and without any obvious motive ; if he affects great courage and resolution, the surgeon should be upon his guard. The slightest excitement should be avoided. The patient should be kept in the most complete repose, away from light, noise, or unnecessary visits, or his symptoms will quickly become more decided. He will soon begin to talk unconnectedly ; at one moment his language will be mild, at another violent ; his loquacity will be incessant. In this state the patient is dangerous to others and himself. In one case M. Helis mentions, a man rose in the middle of the night, and beat many of his companions with his crutch, and would probably have destroyed some of them, if he had not been secured. In some instances, patients have precipitated themselves from a window, or have destroyed themselves in a still more horrible manner.

The most remarkable circumstance in the midst of so much disturbance of the mental faculties, is the tranquil state of the circulation, and the absence of febrile symptoms. The patient is furious, has lost all command of himself ; his face is bathed with sweat, his eyes are unusally brilliant, he cries out vociferously, and might be thought to be labouring under the most ardent phrenzy ; but his pulse is calm and regular, and the state of the skin removes all suspicion of inflammation, It is, in fact, a true mania, differing only from ordinary cases in its duration. M. Helis has rarely seen the attack last longer than five or six days.

The mode of exhibiting it is as simple as it is efficacious. It consists of a few drops of laudanum administered in a cylster. It is this remedy which M. Dupuytren constantly employs, and it is far preferable to every other. Five or six drops of laudanum, given in a small clyster, produce more effect than thrice the quantity taken by the stomach. This fact may be explained by the sympathy which unites the brain with the rectum. As a proof that this sympathetic connexion is not imaginary, we may cite various cases of pains in the head, delirium from constipation, the clear and active state of the mind which follows a required evacuation from the bowels, and instances of hemicrania that have resisted every other treatment, and which have yielded, as if by enchantment, to irritants placed in the rectum. But a physiological explanation may be adduced. The stomach, destined to elaborate the first element of nutrition, is endowed with a digestible power, and with secretions which alter more or less every substance which comes in contact with it ; and many medicines introduced into the stomach are ineffectual, because they are mixed with the food, or their powers are weakened by the gastric juice. Hence various medicines, particularly of the vegetable class, are uncertain in their operation, or totally inefficacious, with many patients. The rectum, destined to be the reservoir of the residue of digestion, absorbs, but does not digest, and it will easily be conceived that medicines which are introduced in it, provided they are not expelled, will act with more certainty than if they were administered by the stomach.

The treatment recommended by M. Helis in cases ef traumatic delirium, has been practised with much success in several of the London hospitals, but we know it is not yet duly appreciated by the profession at large. Two instances have occurred to us in which anodynes had been given by the mouth without benefit. In both, the exhibition of an enema, with fifteen drops of laudanum, speedily quieted the turbulance and incessant loquacity of the patients ; a calm sleep followed, and health was soon restored.—*London Medical and Physical Journal.*

42. *Biographical Notice of Soemmering.*—This distinguished anatomist and physiologist died at Frankfort, March 6th, in the 76th year of his age; and the following notice of his earthly career is contained in the French *Journal Hebdomadaire.*

From the character of his writings, and the solid basis of his reputation, Soemmering may be looked upon as one of the fathers of science. To the Germans, indeed, he appeared in the light of a contemporary and fellow-laborer; but to us, who have only known him in his works, he seems rather to have belonged to another age. We associate the idea of him with that of Albinus, with whom he possessed so much in common.

Samuel Thomas Von Soemmering was born at Thorn, the 25th of January, 1755. He received the degree of Doctor at the University of Gottingen, in April, 1778, and from that period began to establish in Germany that reputation for science which continued to increase with his works. The inaugural dissertation of Soemmering was entitled

Dissertatio de basi Encephali, et Originibus Nervorum Cranio Egredientium." Already, in this first and important work, appeared that admirable activity of investigation, and astonishing power of invention, which always characterized the talent of Soemmering. In 1779 he published a volume, in quarto, on the Functions of the Lymphatic System in Health and Disease, and on the application to be made of such knowledge to the purposes of practical medicine.

About this period, so fruitful in moral and political discussions, many philosophers, and among others, Raynal and Condorcet, were wont to plead the cause of the negroes, whose emancipation they demanded in vehement and systematic declamations. Attention was directed to this question from every quarter, and it was on this occasion that Soemmering published his Treatise on the Physical Differences which distinguish the Negro from the European. The first edition of this work was published at Mayence, in 1784, and was followed by another, which appeared at Frankfort, in 1785. In the same year our author produced a Dissertation on the small Calculi which are found in the Pineal Gland and its immediate vicinity. Always interested about the brain, Soemmering published a work on the Decussation of the Optic Nerves, and in 1788, one on the Brain and Spinal Marrow. In the interval between these two publications, he composed a Memoir on Crises and Critical Derangements. Another, in 1788, made much noise in Germany and France, perhaps chiefly owing to the nature of the subject; it was upon the pernicious Effects of Corsets. The numerous discoveries which he had made on the structure of the brain, were only known to the Savans, but no sooner did he write about stays, than all Europe became familiar with his name!

The cabinet of Cassel contained a magnificent collection of monsters. Soemmering studied with care all the curious examples which were there collected, and, in a treatise on the subject, he described the singular cases which he had remarked in this museum. Even here he contrived to be original, in a description which appeared little calculated for the display of talent. The most able part of these observations is that which relates to acephalous and polycephalous monsters. In 1791 appeared a work on the Cure of Calculus; and in 1795, he composed, in conjunction with T. Wentzel, a very interesting Dissertation on the bones of Gouty Persons.

It was maintained by some, that fracture of the vertebræ was always mortal: Soemmering combatted this opinion; and, in a work which appeared in 1793, he proved, by facts and reasoning thereupon, that even in the cases where chronic disease of the vertebræ has produced their entire destruction, a chance of safety may remain.

We have not yet spoken of one of the works of Soemmering which has obtained the greatest success, and not without cause: it is his Manual of the Structure of the Human Body. A great number of editions, published at different times, attest the merit of the work. In some places, indeed, where the art of multiplying editions has become an integral part of literary merit, this would have been no decisive proof; but in Germany, where they do not recompose a book till the former edition has been sold, the repetition of the publication generally shows that the work is meritorious. The one in question is remarkable for the extreme fidelity of the descriptions, as well as for the number and variety of the facts which it contains. Some parts deserve higher commendation; such are the osteology and the description of the brain and nerves. The last, indeed, was always a favorite subject with Soemmering, and among his later works upon it, was one entitled, "On the Organ of the Soul." In this he maintains an opinion which has not the merit of novelty, nor the solidity of his general doctrines; he holds, namely, that the soul has its seat in the humidity which, during life, lubricates the ventricles of the brain. In 1811 he gave an account of some interesting researches regarding the fluid in the nerves, and on its uses and connexion with the nutrition of these organs in the healthy and diseased conditions. Nor must we omit to mention, in relation to this part of the subject, his tables of the base of the brain, in which are represented with great beauty, and we believe with extreme correctness, the principal differences which exist between the encephalon in man and the lower animals.

The Society of Gottingen had published a programme on the causes and prevention of hernia. Soemmering replied to this in a treatise on Umbilical and Inguinal Ruptures. * * * In the last of these he advanced the opinion now generally adopted, that umbilical hernia never forms in adults through the umbilical cicatrix itself, but in the linea alba in its neighborhood. The works of Soemmering almost defy enumeration in a notice such as this, but we must add to the list his plates of the Ear, the Eye, and Organs of Voice, and those of the Human Embryo. It it this last which has led the way to the important researches in embryology, which have since been carried on by the Germans,—Baer, Meckel, Tiedemann, Carus, and others.—Soemmering, however, was the first who gave an exact figure of the embryo, and of the successive gradations of its development from the fourth week after its conception.

The last work of this illustrious author, was on the Fatal Diseases of the Bladder in Old Persons, the first edition of which appeared in 1809, and a second in 1822, being, as we believe, the close of Soemmering's professional writings.

In 1828, Soemmering attained the 50th year of his doctorate. It is a general usage in Germany, to celebrate a kind of jubilee in honor of those who have grown old in scientific labors and fame. On this occasion, all the most distinguished men in Germany, hastened to render homage to the aged philosopher.—*Bost. Med. and Surg. Jour.*

43. *Obituary of Dr. John Doane Wells.*—It was our intention to have published in this number of the Journal, a sketch of the life of Dr. Godman. Unforseen circumstances have prevented the accomplishment of that object. Shortly after the death of Dr. Godman, the Profession of Medicine sustained an equal loss in the death of John Doane Wells, M. D., recently elected Professor of Anatomy in the University of Maryland, who died on the 25th of July, at the early age of 31 years, in the morning of life, and at the opening of the most brilliant professional prospects, that youthful ambition could aspire to.

He was born March 6th, 1799, gratuated at Harvard College, in 1817, and received the degree of Doctor of Medicine in 1820. In 1821, he was appointed Professor of Anatomy and Surgery in Brunswick College, in Maine. The same year he visited Europe, where he devoted himself particularly to anatomical pursuits. He returned to the United States in December, 1822, and commenced his first course of lectures in February, 1823. In 1826, he was unanimously elected Professor of Anatomy in the Berkshire Medical Institution, at Pittsfield, Mass., and as the course of lectures were to be delivered at a different period of the year, he retained the chair in both institutions.

In the month of October last, he received an intimation of his appointment as Lecturer on Anatomy, in the Maryland University. His course at Pittsfield was not yet completed, although, in anticipation of this appointment, he had been lecturing two, three, and sometimes four times a day. Having been honorably discharged from this institution, he hastened to Baltimore, although exhausted by his severe labour at the former place.

Having completed his course of Lectures in Baltimore, he set out for Brunswick, in very unfavorable weather, and arrived there with every evidence of extreme debility from over exertion. In spite of the remonstrances of his friends, he persisted in lecturing. He would leave his bed to be carried to the college, and having delivered his lecture, be carried home again, and keep his bed for the remainder of the day. In a week he was compelled to discontinue his labors. His constitution had received a shock from which he never recovered, and after great and protracted suffering, he died in July.

We copy from the Boston Med. and Surg. Journal, the following account of his moral and intellectual character, regretting that it is not in our power to insert the whole memoir:

"The friends of Dr. Wells do not claim for him uncommon greatness or originality of mind ; although all must admit that his talents were of a high order. He was particularly distinguished for quickness of perception; and if not calculated to strike out new paths in the fields of science, he was ready in discerning and embracing important truths, when once presented to his mind. His memory was ready and retentive, and the correctness of his judgment generally led him to the adoption of just opinions. He had rendered himself well acquainted with the most approved writers in our profession. His mind was not crowded with a knowledge of useless and obsolete opinions, but it was well stored with useful learning, which he possessed the happy faculty of turning, on fit occasions, to a good account. As a lecturer, we are not able to speak of him from personal knowledge. Those who have known him in that capacity, speak of him as possessing great powers His voice was clear and strong, and his utterance distinct; his countenance animated and full of expression; his command of language uncommonly great; his acquaintance with his subject very thorough; and his active and collected mind wielded with great skill the materials with which it was enriched. His zeal in his profession was unquenchable, and it urged him on with a power, which neither languor, nor pain, nor sickness, could in any degree subdue. Such was his love for lecturing, that during the intermissions, in his visits to his friends in this city, he would often express to them a longing desire for the time to return when he could engage in it.

It was his moral qualities united to his intellectual powers which constituted the charm of his character. It was these that rendered him one of the most interesting of men; that endeared him so much to his friends, and won the confidence and esteem of all who became acquainted with him. It was these qualities that made him the good son; the affectionate brother; the faithful friend and the pleasing companion, and which cause his death to be so keenly felt through a large circle of acquaintance. In his disposition Dr. Wells was uncommonly benevolent and affectionate. An unkind word never escaped his lips; an unkind feeling never found a place within his bosom. He was susceptible of all the tender sympathies of life. He knew how to impart happiness to his friends, and to cheer the drooping spirits of the downcast stranger.

Should we be asked what was the most prominent trait in his character, we should be unable to answer. No one quality stood pre-eminent beyond the rest, neither was there any striking defect which his friends had to lament as detracting from the beauty of the whole. His mind was remarkably well balanced, and presented every part in beautiful and harmonious proportions. In his domestic relations his duties were performed with exemplary fidelity; his friendship was ardent, sincere, and generous; his benevolence free from ostentation, and tempered with judgment; and his intercourse with the world was marked with honor and integrity. His mind was well disciplined, and his appetites, passions, and affections, were subjected to the higher powers of reason and conscience, and rendered subservient to the great purposes of his moral and intellectual being. His learning was free from pedantry, and amidst all the caresses and flatteries of his numerous friends and admirers, he retained the native modesty of his disposition. Some may be inclined to attribute his success in life to fortuitous circumstances; but such was the activity of his spirit and the energy of his purpose, that, under circumstances much less propitious, he would have attained to distinction. He was ambitious,—but his was not that sordid ambition which terminates in self-aggrandizement and self-promotion; it was of that lofty and honorable character which aims at acting well and promoting the great interests of society.

From what has already been said, it might be inferred that the mind of our friend was no stranger to those holy influences and affections which unite man to his Maker, and fit him for the invisible world. His religious principles were not the mere result of habit and early education, but were founded in the honest convictions of his mind. Religion was with him a practical principle which had become intimately blended with his soul, and which purified and sanctified all his affections. It was this principle that supported him in life, and sustained him in death. It was this that preserved him uncorrupted amidst the flatteries and enticements of his prosperous course; and it was this that supported him under the trials and sufferings of a lingering sickness. There were periods during the confinement which preceded his death, in which his character exhibited a moral sublimity which it was delightful to witness.

Such was the life and such the death of our valued friend. No one ever had greater cause for wishing to live, and no one could be more ready to die. During his life he was the comfort and pride of his friends, and in his death he has left them full of the hope and consolation that he has gone to inherit a crown of everlasting glory."

